# Cell membrane vesicles derived from hBMSCs and hUVECs enhance bone regeneration

**DOI:** 10.1038/s41413-024-00325-9

**Published:** 2024-04-09

**Authors:** Dandan Wang, Yaru Guo, Boon Chin Heng, Xuehui Zhang, Yan Wei, Ying He, Mingming Xu, Bin Xia, Xuliang Deng

**Affiliations:** 1 Department of Pediatric Dentistry, Peking University School and Hospital of Stomatology, Beijing, 100081 P. R. China; 2 Department of Geriatric Dentistry, Peking University School and Hospital of Stomatology, National Center for Stomatology, National Clinical Research Center for Oral Diseases, National Engineering Research Center of Oral Biomaterials and Digital Medical Devices, Beijing, 100081 P. R. China; 3 Department of Dental Materials & Dental Medical Devices Testing Center, Peking University School and Hospital of Stomatology, National Engineering Research Center of Oral Biomaterials and Digital Medical Devices, Beijing, 100081 P. R. China

**Keywords:** Bone, Bone quality and biomechanics

## Abstract

Bone tissue renewal can be enhanced through co-transplantation of bone mesenchymal stem cells (BMSCs) and vascular endothelial cells (ECs). However, there are apparent limitations in stem cell-based therapy which hinder its clinic translation. Hence, we investigated the potential of alternative stem cell substitutes for facilitating bone regeneration. In this study, we successfully prepared cell membrane vesicles (CMVs) from BMSCs and ECs. The results showed that BMSC-derived cell membrane vesicles (BMSC-CMVs) possessed membrane receptors involved in juxtacrine signaling and growth factors derived from their parental cells. EC-derived cell membrane vesicles (EC-CMVs) also contained BMP2 and VEGF derived from their parental cells. BMSC-CMVs enhanced tube formation and migration ability of hUVECs, while EC-CMVs promoted the osteogenic differentiation of hBMSCs in vitro. Using a rat skull defect model, we found that co-transplantation of BMSC-CMVs and EC-CMVs could stimulate angiogenesis and bone formation in vivo. Therefore, our research might provide an innovative and feasible approach for cell-free therapy in bone tissue regeneration.

## Introduction

Angiogenesis and osteogenesis are two key elements in bone formation.^1–3^ Promoting the vascularization of bone is a key strategy for achieving favorable bone defect healing. Recent studies have demonstrated that stem cell-based therapies have great potential in bone regeneration therapies.^4–6^ Mesenchymal stem cells (MSCs) have the ability to generate mineralized tissues and are involved in the process of bone formation, which make them a crucial cell type in bone regeneration.^7,8^ Additionally, vascular endothelial cells (ECs) can enhance the osteogenic differentiation and regenerative potential of MSCs through paracrine signaling.^9,10^ Co-transplantation of MSCs and ECs into bone defects to achieve vascularization and bone formation respectively, is an effective therapeutic strategy for achieving bone tissue repair.^11^

Despite the significant advantages of stem cell therapy, there still exists some limitations. Ethical controversies and risks of tumor formation limit the widespread clinical applications of stem cell-based therapy.^12–14^ Additionally, cells transplanted into the body are susceptible to the micro-environment that affects their self-renewal and differentiation potential, even leading to the secretion of cytokines detrimental to tissue regeneration, such as pro-inflammatory mediators.^15^ Furthermore, the relatively large size of cells impedes their mobility in blood vessels and tissues, consequently limiting their applications in targeted therapy.^16^ Therefore, the exploration of stem cell substitutes is a highly promising research direction in tissue regeneration. Here, we focused on cell membrane vesicles (CMVs) induced by cytochalasin B (CB), which display favorable bioactivity and are amenable to large-scale manufacturing.^17,18^ CB is known as an effective chemical agent for destabilizing cytoskeleton-membrane interactions. CMVs were prepared after treatment with CB and were released from the cell surface through mechanical shear forces. This method results in the orientation and functional activity of cell surface receptors, ion pumps and cytosolic proteins being well preserved in CMVs.^17^

**Fig. 1:**
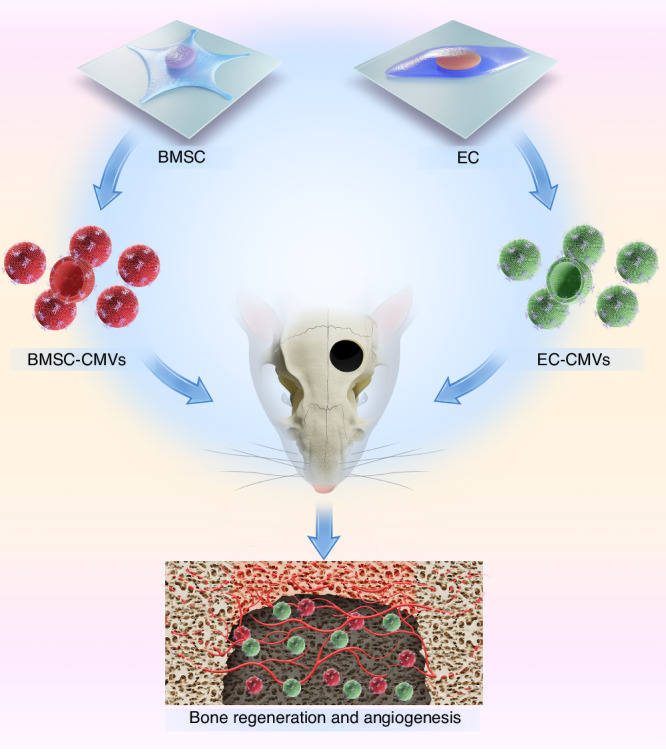
Schematic illustration of BMSC-CMVs and EC-CMVs preparation, together with their application in vivo

CMVs have the following advantages including a wide diverse variety of cell sources, simple preparation methods, short preparation time and high yield, which confer great clinical application potential.^19,20^ In theory, all eukaryotic cell types are amenable to CB treatment since they all possess cellular actin cytoskeleton.^21^ Because CMVs can maintain the functional activity of surface receptors and cytosolic proteins derived from their parent cells, they have been widely investigated in cellular signaling and communication in recent years.^22,23^ However, the effects and underlying mechanisms by which the combination of BMSC-derived CMVs and EC-derived CMVs promote bone regeneration are still largely unknown. Here, we successfully prepared human bone marrow mesenchymal stem cells derived CMVs (BMSC-CMVs) and human umbilical vein endothelial cells derived CMVs (EC-CMVs), which contain biologically active molecules derived from their parental cells (Fig. 1). We found that BMSC-CMVs could enhance the tube formation and migration ability of hUVECs and EC-CMVs could enhance the osteogenic differentiation of BMSCs in vitro. The combined application of BMSC-CMVs and EC-CMVs synergistically promoted bone tissue repair in rat skull defects, thus suggesting that this might be a promising cell-free therapeutic strategy for facilitating bone regeneration.

## Results

### Preparation and characterization of BMSC-CMVs and EC-CMVs

**Fig. 2:**
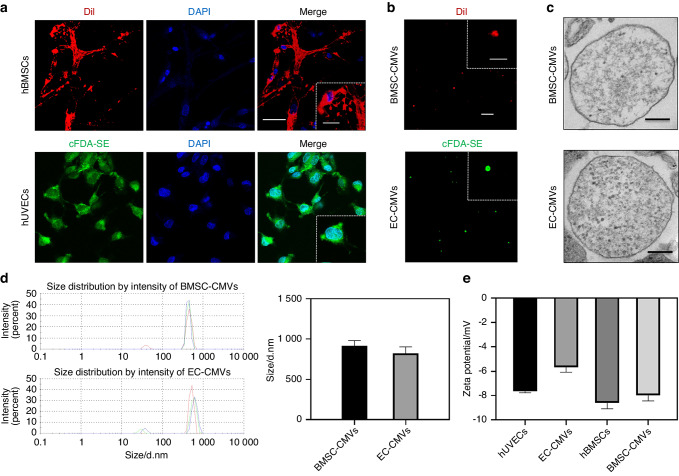
The preparation and characterization of BMSC-CMVs and EC-CMVs. **a** Representative images of DiI labeled hBMSCs and cFDA-SE labeled hUVECs treated with cytochalasin B for 30 min (the scale bar = 50 μm), with insets showing a higher magnification (the scale bar = 20 μm). **b** Representative CLSM images of BMSC-CMVs and EC-CMVs (the scale bar = 20 μm), with insets showing higher magnification images (the scale bar = 5 μm). **c** Representative TEM images of BMSC-CMVs and EC-CMVs. Scale bars = 200 nm. **d** The size distribution of BMSC-CMVs and EC-CMVs. The peaks analyzed by DLS showed that the average sizes of BMSC-CMVs and EC-CMVs were approximately 916.5 nm and 822.3 nm (d, diameter) respectively (*n* = 3). **e** The surface ζ-potential of hBMSCs, hUVECs, BMSC-CMVs and EC-CMVs (**P < 0.01, *n* = 3)

Human bone marrow mesenchymal stem cells (hBMSCs) and human umbilical vein endothelial cells (hUVECs) were purchased from WUHAN PROCELL LIFE SCI&TECH CO., LTD and the characterization and validation of these two human primary cells are presented in Figs. S1 and S2. Then CMVs were obtained from hBMSCs and hUVECs. After treatment with cytochalasin B (CB) for 30 min, the actin microfilaments of cells were disrupted and spherical cell capsules were observed around hBMSCs and hUVECs (Fig. 2a and Fig. S3). The rounded membrane-enclosed vesicles were then separated from cells and subsequently collected. Next, fluorescent BMSC-CMVs and EC-CMVs were obtained from DiI labeled hBMSCs and cFDA-SE labeled hUVECs, respectively (Fig. 2b). The morphology and structural properties of CMVs were analyzed by TEM (Fig. 2c). CLSM and TEM images showed that BMSC-CMVs and EC-CMVs were composed of intact membrane and cytoplasmic structures, which were derived from their parental cells. The sizes were analyzed by dynamic light scattering (DLS) and the results indicated that the average sizes of BMSC-CMVs and EC-CMVs were 916.5 nm and 822.3 nm, respectively (Fig. 2d). Additionally, although there was a statistically significant difference between the Zeta potentials of hUVECs and EC-CMVs, the BMSC-CMVs and EC-CMVs possessed similar slightly negative surface charges as hBMSCs and EC-CMVs respectively, indicating that CMVs have similar characteristics as their parental cells (Fig. 2e).

### The BMSC-CMVs enhanced tube formation and migration of hUVECs in vitro

**Fig. 3:**
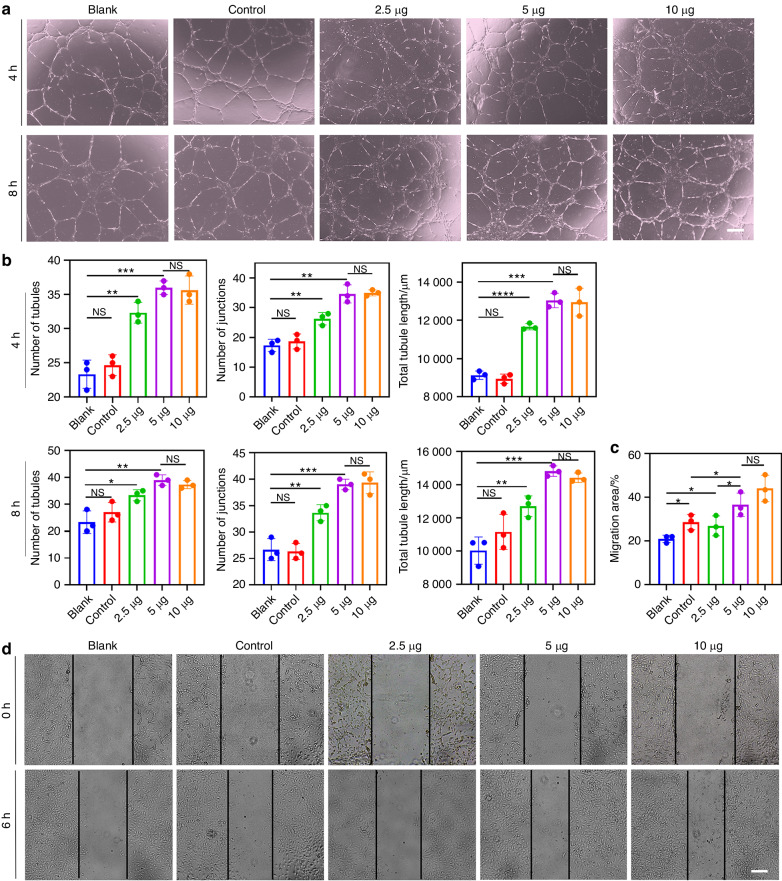
BMSC-CMVs enhanced tube formation and migration of hUVECs in vitro. **a** Representative images of tube formation at 4 and 8 h after hUVECs were seeded on Matrigel. hUVECs co-cultured with conditioned medium of hBMSCs were used as a blank control; untreated hUVECs were used as a blank. The scale bar = 200 μm. **b** Number of tubules, number of junctions and total tubule lengths in (**a**) at 4 and 8 h were quantified using Image Pro Plus 6.0 software (*P < 0.05, **P < 0.01, ***P < 0.001, ****P < 0.000 1, *n* = 3). **c** The quantitative migration area analysis of hUVECs in (**d**) (*n* = 3). **d** Scratch wound assay of hUVECs. The scale bar = 200 μm

To assess the pro-angiogenic effects of BMSC-CMVs on hUVECs, hUVECs were co-cultured with BMSC-CMVs in different quantities (2.5, 5, 10 μg) for 4 h and 8 h, respectively. The number of tubules, junctions and tubule lengths of hUVECs in the co-culture group were significantly increased compared to hUVECs in the blank and control groups (Fig. 3a, b). These results indicated that the BMSC-CMVs could enhance tube formation of hUVECs in vitro and that tube formation would not be enhanced further when the quantity reached 5 μg. Then the effects of BMSC-CMVs on the migration capacity of hUVECs were detected using a scratch wound assay. The migration area of hUVECs was markedly increased in the BMSC-CMVs groups compared to that in the blank group at 6 h, indicating that co-culture with BMSC-CMVs markedly enhanced the mobility of hUVECs (Fig. 3c, d). However, the migration area would not be enhanced further when the quantity reached 5 μg, and the migration ability of hUVECs in the 5 μg group were stronger than that in the control group (Fig. 3c, d). Collectively, these results thus demonstrated that BMSC-CMVs could enhance the tube formation and migration ability of hUVECs in vitro.

### EC-CMVs promoted the osteogenic differentiation of hBMSCs in vitro

**Fig. 4:**
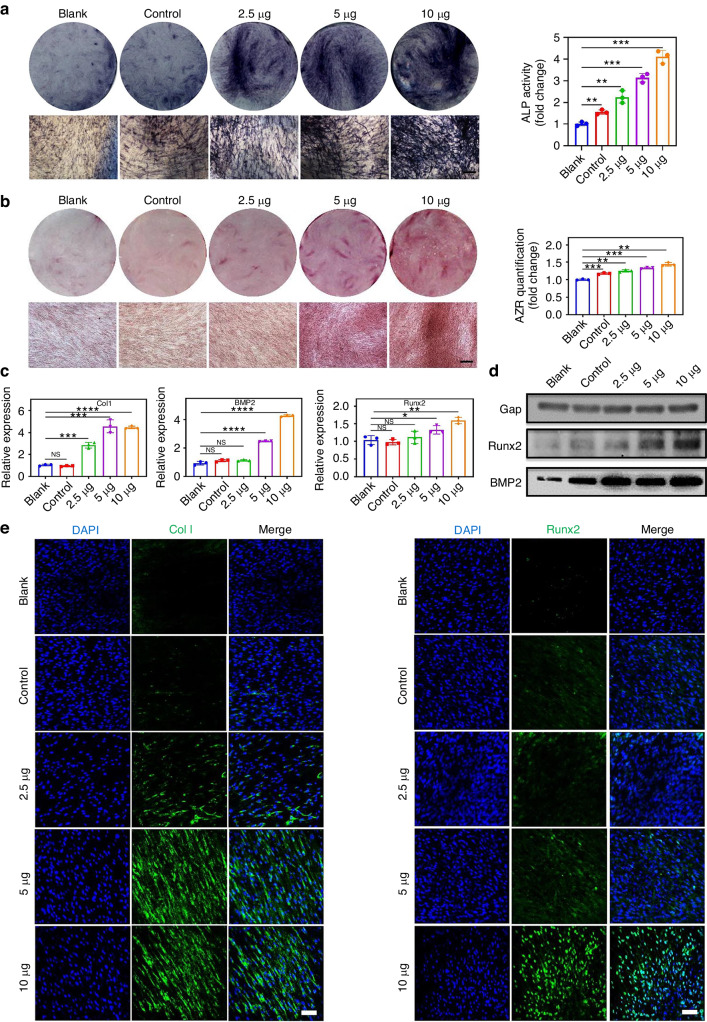
The pro-osteogenic effects of EC-CMVs on hBMSCs. **a** Alkaline phosphatase staining and quantification of cells at 7 days after co-culture; hBMSCs co-cultured with conditioned medium of hUVECs were used as a blank control; hBMSCs without treatment were used as a blank (**P < 0.01, ***P < 0.001, *n* = 3). The scale bar = 500 μm. **b** Alizarin red staining and quantification of cells at 14 days after co-culture. The scale bar = 500 μm. **c** Gene expression levels of hBMSCs after different treatments at 7 days in vitro (*P < 0.05, ****P < 0.000 1, *n* = 3). **d** Runx2 and BMP2 protein expression levels of hBMSCs after different treatments at 7 days in vitro. **e** Immunofluorescence staining for detection of ColI and Runx2 by hBMSCs after different treatments at 7 days in vitro. Scale bars = 100 μm

To evaluate the pro-osteogenic effects of EC-CMVs on hBMSCs, hBMSCs were co-cultured with EC-CMVs in different quantities (2.5, 5, 10 μg) for 1 week and 2 weeks, respectively. In the co-culture group, increased alkaline phosphatase (ALP) activity and calcium nodule formation of hBMSCs were observed after co-culture with EC-CMVs, with more calcium mineralization as the proportion of hBMSC increased (Fig. 4a, b). Reverse transcription polymerase chain reaction (RT-PCR) showed that the mRNA levels of ColI, BMP2 and Runx2 were significantly higher in the co-culture group versus the blank and control groups (Fig. 4c). The Runx2 and BMP2 protein expression levels in the co-culture group were much higher than the blank group, as determined by western blots (Fig. 4d and Fig. S4). Furthermore, immunofluorescence staining images for detection of both Runx2 and ColI proteins showed that fluorescence intensities in the hBMSCs of the co-culture groups were much higher compared to the hBMSCs of the blank group (Fig. 4e). Collectively, these results suggested that co-culture with EC-CMVs enhanced the osteogenic differentiation of hBMSCs in vitro, with the quantity of 10 μg, yielding the highest osteogenic activity of hBMSCs.

### Mechanistic analyses of pro-osteogenic and pro-angiogenic effects mediated by CMVs

**Fig. 5:**
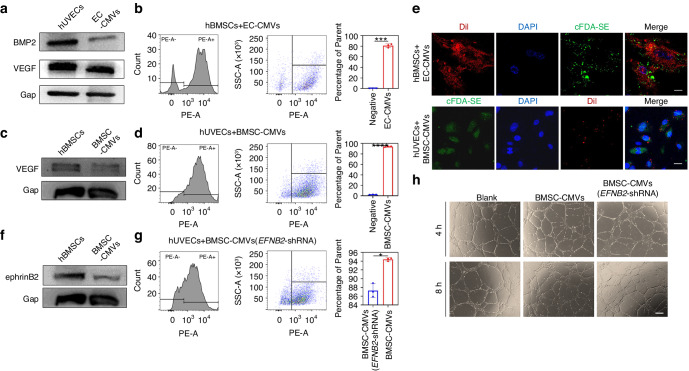
The mechanistic analysis of pro-osteogenic and pro-angiogenic effects mediated by CMVs. **a** Representative western blots showed that EC-CMVs contained BMP2 and VEGF, similar to their parental cells. **b** The endocytosis of EC-CMVs by hBMSCs was analyzed by flow cytometry (***P < 0.001, *n* = 3). **c** Representative western blots showed that BMSC-CMVs contained VEGF, similar to their parental cells. **d** The endocytosis of BMSC-CMVs by hBMSCs was analyzed by flow cytometry (****P < 0.000 1, *n* = 3). **e** EC-CMVs fluorescence emission (green) were observed in hBMSCs. 4′,6-diamidino-2-phenylindole (DAPI)-labeled cell nuclei are indicated by blue fluorescence. BMSC-CMVs fluorescence emission (red) were observed in hUVECs. Scale bars = 20 μm. **f** Representative western blots showed that BMSC-CMVs contained ephrinB2, similar to their parental cells. **g** The endocytosis of BMSC-CMVs and BMSC-CMVs (EFNB2-shRNA) by hUVECs respectively were analyzed by flow cytometry (*P < 0.05, *n* = 3). **h** Representative images of tube formation at 4 and 8 h after hUVECs were seeded on Matrigel and co-cultured with BMSC-CMVs and BMSC-CMVs (*EFNB2*-shRNA) respectively; untreated hUVECs were utilized as a blank. The scale bar = 200 μm

In our study, we found that CMVs derived from hUVECs and hBMSCs could promote osteogenesis and angiogenesis respectively in vitro. CMVs could be considered as potential mediators of cell-to-cell communication. Accordingly, we investigated the roles that BMSC-CMVs and EC-CMVs played in tube and bone formation in vitro. The protein composition of CMVs derived from hUVECs were determined by western blots and the results showed that EC-CMVs contained bone morphogenetic protein-2 (BMP2) and vascular endothelial growth factor (VEGF), similar to their parental cells, which are key growth factors in the osteogenic process (Fig. 5a and Fig. S5). The flow cytometry results and immunocytochemical staining images showed that EC-CMVs were engulfed by hBMSCs after co-culture for 24 h (Fig. 5b, e). Hence, these results demonstrated that EC-CMVs contained proteins that are beneficial for improving the bone formation, and that endocytosis of EC-CMVs by hBMSCs enhanced their osteogenic activity.

Likewise, the western blots showed that BMSC-CMVs contained the key angiogenic cytokine, VEGF, which plays a crucial role in the angiogenesis process (Fig. 5c and Fig. S6). The flow cytometry results and immunocytochemical staining images showed that BMSC-CMVs were engulfed by hECs after co-culture for 24 h (Fig. 5d, e). According to previous studies, the forward ephrinB2/Ephs signaling from hBMSCs could enhance the tube formation activity of hUVECs.^24^ In our research, we detected ephrinB2 expression on BMSC-CMVs, similar to their parental cells (Fig. 5f and Fig. S7). To confirm the pro-angiogenic effects of ephrinB2/Ephs signaling in the interaction between hUVECs and BMSC-CMVs, we downregulated ephrinB2 expression in BMSC-CMVs by knocking down *EFNB2* expression in hBMSCs using *EFNB2*-targeting small-hairpin RNAs (Fig. S8). The results of flow cytometry and immunocytochemical staining indicated that hECs engulfed fewer BMSC-CMVs after downregulation of ephrinB2 expression in BMSC-CMVs (Fig. 5g, e and Fig. S10). Then the total tubule length, number of tubules and junctions of hUVECs co-cultured with BMSC-CMVs (*EFNB2*-shRNA) was observed to be decreased after ephrinB2 expression was downregulated in BMSC-CMVs (Fig. 5h and Fig. S9). To further verify the angiogenic effects of ephrinB2/Ephs signaling from BMSC-CMVs to hUVECs in vivo, we transplanted hUVECs with BMSC-CMVs and BMSC-CMVs (*EFNB2*-shRNA) in nude mice by subcutaneous injection. The immunohistochemical staining images showed that the CD31 positive area was significantly increased in the hUVECs+BMSC-CMVs group compared to the hUVECs group, thus indicating that BMSC-CMVs could enhance the angiogenesis of hUVECs in vivo (Fig. S11). Moreover, the blood vessel area was decreased in the hUVECs+BMSC-CMVs (*EFNB2*-shRNA) group compared to that in the hUVECs+BMSC-CMVs group (Fig. S11). Collectively, these results confirmed that growth factors contained in BMSC-CMVs could promote the pro-angiogenic effects of hUVECs, and that ephrinB2/Ephs signaling from BMSC-CMVs to hUVECs could enhance the tube formation activity of hUVECs.

Additionally, a more comprehensive technique, namely proteomics, was used to further screen the genes and proteins related to osteogenesis and angiogenesis, as well as analyze the various functional molecules and signaling pathways of EC-CMVs and BMSC-CMVs respectively. The results showed that EC-CMVs and BMSC-CMVs abundantly expressed various genes related to osteogenesis and angiogenesis respectively (Tables S1, 2). The results of KEGG (Kyoto Encyclopedia of Genes and Genomes) analysis presented in Figs. S12 and S13 showed that BMSC-CMVs and EC-CMVs both possess a wide array of protein components implicated in numerous diverse biological processes, such as cellular processes, metabolism and organismal systems. The G.O (Gene Ontology) enrichment analysis showed that BMSC-CMVs contain proteins originating form various cellular components including plasma membrane, cytosol and cytoplasm. These proteins are associated with numerous diverse biological processes, including angiogenesis, signal transduction, cell growth, cell adhesion, protein metabolism, cell migration and wound healing (Fig. S14). The EC-CMVs also contain proteins from various cellular components including cytosol, cytoplasm and plasma membrane. These proteins are also implicated in a variety of biological processes, including signal transduction, cell adhesion, cell differentiation, and cell adhesion (Fig. S15), and exhibit a diverse array of molecular functions, such as protein binding, ion binding and integrin binding. Collectively, these results thus indicate that CMVs have much potential in tissue engineering.

### Implantation of BMSC-CMVs and EC-CMVs in vivo stimulated angiogenesis and bone formation

**Fig. 6:**
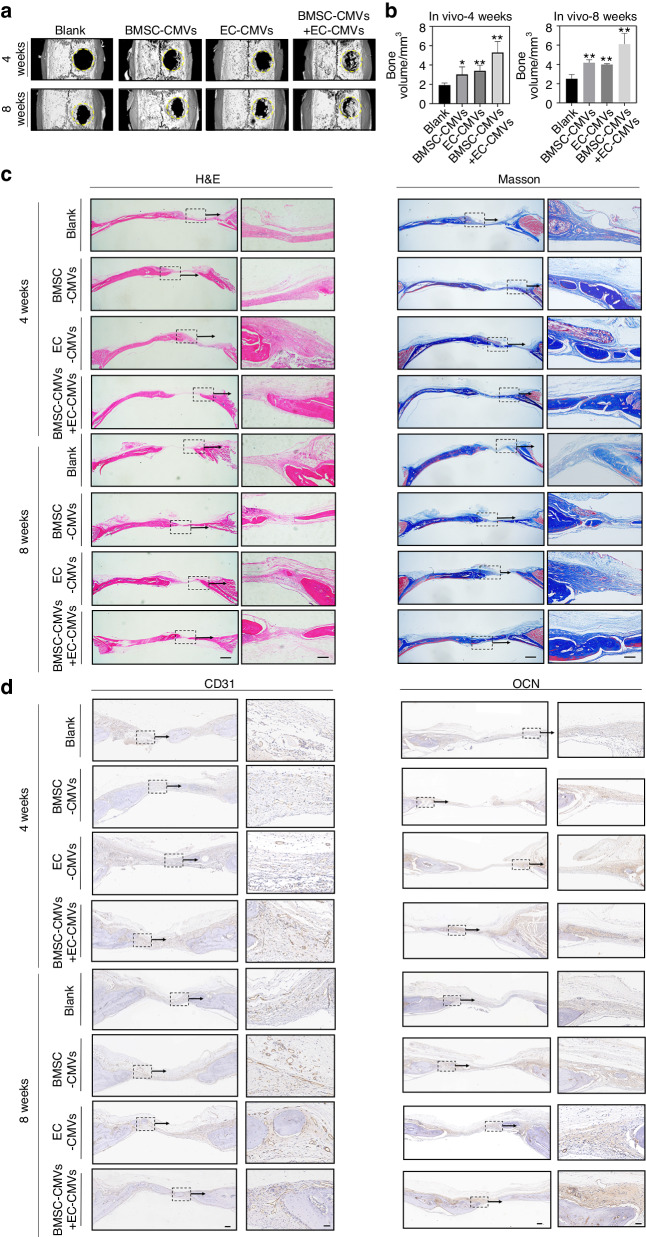
CMVs stimulated angiogenesis and bone regeneration in vivo. **a** Representative micro-CT images of rat calvaria defects and bony islands at 4 weeks and 8 weeks post-implantation, respectively. Rat calvaria defects were treated with Matrigel mixed with BMSC-CMVs, EC-CMVs and BMSC-CMVs+EC-CMVs, respectively. Matrigel treated rats were assigned as the blank group. **b** Quantitative analysis of bone volume (mm^3^) in the defect regions. Statistics were analyzed based on μCT analysis (*P < 0.05 and **P < 0.01 compared with the blank group, *n* = 6). **c** Hematoxylin/eosin and Masson staining images of the calvaria defects in the four groups after 4 weeks and 8 weeks post-implantation respectively (The scale bar = 1 mm). The images on the right showed a higher magnification of the boxed region in the images on the left (The scale bar = 200 μm). **d** Immunohistochemical staining images for detection of CD31 and OCN expression within the calvaria defects of the four groups at 4 and 8 weeks post-implantation respectively (The scale bar = 200 μm). The images on the right showed a higher magnification of the boxed region in the images on the left (The scale bar = 50 μm)

To confirm whether CMVs could stimulate angiogenesis and bone formation in vivo, we implanted CMVs derived from rat BMSCs and ECs into the rat skull defects. At week 4 and 8 post-surgery, bone formation in the calvarial defects were evaluated. As assessed by microcomputed tomography (micro-CT), treatment with BMSC-CMVs or EC-CMVs mixed in Matrigel improved the volume of the newly-formed bone within the calvarial defects compared to the blank group, while the bone volume in the co-transplantation of BMSC-CMVs with EC-CMVs group was significantly higher than the other 3 groups (Fig. 6a, b). Histological and Masson staining images revealed that after co-transplantation of BMSC-CMVs and EC-CMVs for 8 weeks, the bone defects were filled with mainly contiguous bone structures, whereas for the blank group only fibrous scar tissues were observed (Fig. 6c). The immunohistochemical staining images showed that co-transplantation of BMSC-CMVs with EC-CMVs promoted slightly upregulated expression of the canonical osteoblast marker Osteoclacin (OCN) at 4 weeks post-surgery, and significantly enhanced OCN expression at 8 weeks post-surgery (Fig. 6d and Fig. S16). Hence, these results indicated that BMSC-CMVs and EC-CMVs promoted the repair of calvaria bone defects and restored bone structural integrity. Bone vasculature plays an essential role in bone repair and remodeling, thus blood vessel formation is crucial both in the process of early bone construction as well as in later bone maturation. Consistent with our data in vitro, the immunohistochemical staining showed that the total cross-sectional area of blood vessels increased in the BMSC-CMVs and EC-CMVs co-transplantation group, thus indicating that BMSC-CMVs and EC-CMVs substantially improved blood vessel formation in skull defects (Fig. 6d and Fig. S16). Collectively, these data confirmed that BMSC-CMVs and EC-CMVs could promote osteogenesis and lead to bony tissue formation in vivo.

## Discussion

Bone tissue regeneration and healing are complicated processes based on the complex interaction between osteogenesis and angiogenesis. A number of studies suggested that EVs contain biologically active molecules derived from their parental cells and can therefore be utilized as cell-free replacements in tissue regeneration therapy.^25,26^ Reportedly, the transplantation of BMSC-exosomes has been demonstrated to promote osteogenesis and angiogenesis, probably through activation of the BMP2/Smad1/Runx2 and the HIF-1α/VEGF signaling pathways.^27^ Similar to extracellular vesicles, CMVs with natural membrane and cytoplasm could evade recognition of the immune system and are considered as potential delivery systems for drugs and active molecules.^18,28^ Based on our research, CMVs are composed of membrane structure and cytosolic fluids derived from their parental cells and the average size of BMSC-CMVs and EC-CMVs were 916.5 nm and 822.3 nm, respectively, which are consistent with previous studies.^29,30^ Biologically active surface receptors and ion pumps as well as cytosolic proteins are well preserved in such CB-induced vesicles.^17^ Moreover, the production of CMVs is much easier than extracellular vesicles and CMVs could be prepared in large quantities, which make it a promising therapeutic approach for broader applications in the future.

The proliferation, migration and tube formation of endothelial cells contribute to angiogenesis in bone defects, and angiogenesis is one of the key essential elements of bone formation and development.^11,31^ In this study, the results showed that BMSC-CMVs could promote migration and tube formation of endothelial cells in vitro, thus indicating that BMSC-CMVs might enhance angiogenesis in bone defect healing. Blood vessel formation is an indispensable part in bone formation and new blood vessels and angiogenic factors could significantly accelerate the bone healing process.^32^ Vascular scaffolds afford sufficient nutrients, growth factors and oxygen, which can maintain cell viability during physiological development and bone regeneration.^24^ Vascular endothelial growth factor (VEGF) plays a crucial role in angiogenesis. VEGF induces endothelial cell migration and stimulates blood vessel and bone formation through paracrine signaling pathways.^33^ CMVs could preserve intact membranes and cytoplasmic inclusions and can be considered as almost identical to their parental cells, except without having a nuclei.^34^ Our results thus suggest that BMSC-CMVs contained endogenous substances of their parental cells such as VEGF, and BMSC-CMV were observed to be engulfed by hUVECs after co-culture. Accordingly, we postulated that angiogenesis mechanisms mediated by BMSC-CMVs involved various modes of cell signaling transduction, such as exchanges of cytokines between cells and CMVs via paracrine pathways.

Cell surface receptors and ion pumps are well preserved in such CB-induced vesicles. Hence, CMVs can open up various possibilities of exploring cellular communication involving receptor-mediated signaling.^17^ The interaction of surface proteins could bind cells and CMVs together and induce membrane fusion and subsequent heterophilic interaction.^35^ Moreover, a previous study showed that the membrane-binding proteins ephrinB2 and Ephs promoted hUVECs angiogenesis through juxtacrine signaling between hBMSCs and hUVECs.^24^ EphrinB2 and EphB4 which form bidirectional signals in direct cell contact process are both transmembrane proteins. In this study, the western blot results showed that ephrinB2 was expressed on the membrane of BMSC-CMVs, which was similar to their parental cells. Then we knocked down *EFNB2* expression in hBMSCs and obtained BMSC-CMVs with lower ephrinB2 protein expression. These results thus suggested that the tube formation ability of hUVECs was decreased after treatment of BMSC-CMVs with lower doses of ephrinB2 compared to BMSC-CMVs, demonstrating that another possible mechanism of angiogensis might be the juxtacrine interactions between BMSC-CMVs to hUVECs via ephrinB2/Ephs signaling. Besides, CMVs were prepared from their parental cells and this process involves membrane budding and fussion. Our results further confirmed that these cell surface receptors still retain their original cellular location, orientation and function after isolation from their parental cells.

Previous studies have shown that endothelial cells can modulate the differention potential of mesenchymal stromal cells both via direct contact and through paracrine signaling.^36^ In this study, the results suggested that EC-CMVs promoted osteogenetic differentiation of BMSCs and that the expression of osteogenesis-related markers at both the mRNA and protein levels were significantly increased after co-culture with EC-CMVs, thus demonstrating that EC-CMVs could directly enhance the bone formation process. Furthermore, EC-CMVs contained growth factors-VEGF and BMP2, derived from their parental cells, which is a possible osteogenesis mechanism meditated by EC-CMVs. The application of CMVs could avoid the potential adverse effects and limitations of stem cells as well as maintain the accumulation of local cytokines, which are key components of the bone regeneration process. Yet our results only partially revealed the tissue regeneration and restoration mechanisms mediated by CMVs and the underlying complex mechanisms of this process still need further exploration.

CMVs could transfer proteins between hBMSCs and hUVECs, thus altering the gene expression and bioactivity of recipient cells. Additionally, implantation of CMVs instead of cells would avoid immunological reaction and safety issues associated with transplanting live cells into bone defects. During the healing process, the administration of CMVs could maintain the local molecular concentration within the defects and the amount of CMVs is controllable, which can guarantee the stability, safety and efficacy of the repair process. In this study, we prepared cell membrane vesicles from hBMSCs and hUVECs and investigated their effects on bone and blood vessel formation. The in vitro results showed that CMVs derived from hBMSCs could facilitate the angiogenic differentiation and migration of hUVECs, and that CMVs derived from hUVECs could promote osteogenic differentiation of hBMSCs. The success of bone generation is based on the vital role of vascularization, which can provide sufficient nutrients, oxygen and growth factors for bone tissue regeneration process and remove waste and carbon dioxide from the healing areas. The in vivo results indicated that co-implantation of BMSC-CMVs and EC-CMVs markedly increased vascular and bone formation in a rat model of skull defect. Collectively, these results indicated that CMVs can play a key role in mediating the interaction between hBMSCs and hUVECs, which in turn synergistically enhanced bone defect healing in vivo. Hence, CMVs derived from hBMSCs and hUVECs might be applied to tissue regeneration, thereby providing a novel alternative cell-free therapeutic modality.

## Materials and methods

### Cell culture

Primary human bone marrow stromal cells (hBMSCs) and primary human umbilical vein endothelial cells (hUVECs) were purchased from ScienCell Research Laboratories and cultured in mesenchymal stem cell medium (MSCM; ScienCell Research Laboratories, USA) and endothelial cell medium (ECM; ScienCell Research Laboratories, USA) respectively, within a humidified 5% CO_2_ incubator at 37 °C. The cultured cells between passages 3 and 6 were then used in following experiments.

### Preparation and characterization of CMVs

HBMSCs and hUVECs were utilized upon reaching 90% confluence within a 10 cm culture dish and were washed three times with phosphate buffered saline solution (PBS) (pH 7.4), followed by incubation in 3 mL of serum-free MSCM and ECM containing 10 μg/mL Cytochalasin B (Solarbio, beijing, China) for 30 min (37 °C, 5% CO2), respectively. At the end of the incubation, cells formed CMVs and were treated with 0.25% (w/v) trypsin and 0.01% (w/v) ethylenediaminetetraacetic acid (EDTA) at 37 °C for 1 min. Then, the trypsin was inactivated by adding the same volume of FBS and the detached cells were vigorously vortexed for 30 s to separate the cells and newly-formed CMVs. The CMVs were separated from cells by two sequential centrifugation steps (5 min at 200 *g* and 20 min at 2 000 *g*). The BMSC-CMVs and EC-CMVs were collected and fixed with 2.5% (v/v) glutaraldehyde for 1 h. After CMVs were fixed for 1.5 h in 1% (v/v) osmium tetroxide at room temperature, the CMVs were dehydrated in a graded series of ethanol solutions (30, 70, 95 and 100 vol %, respectively) and propylene oxide. Then the samples were embedded in Durcupan (SigmaAldrich) and ultrathin sections were mounted on nickel grids and stained with uranyl acetate. Then the morphology of the BMSC-CMVs and EC-CMVs were observed under transmission electron microscopy (TEM). Then, the hBMSCs were stained with 10 μg/mL DiI (Solarbio, Beijing, China) at 37 °C for 15 min, while the hUVECs were stained with 10 μg/mL CFDA-SE (Carboxyfluorescein diacetate, succinimidyl ester, Solarbio, Beijing, China) at 37 °C for 1 h. The fluorescent-labeled BMSC-CMVs and EC-CMVs were obtained from fluorescent-labeled hBMSCs and hUVECs respectively and observed under laser scanning confocal microscopy (CLSM, Leica). The size and ζ- potential measurement of CMVs were measured by dynamic light scattering (DLS) at room temperature using Malvern Zetasizer nano ZS.

### Alkaline phosphatase (ALP) staining

In this study, CMVs could be regarded as a sphere, thus their volume was estimated by the diameter of the sphere and their density defaulted to 1 g/m^3^. To assess the pro-osteogenic effects of EC-CMVs on hBMSCs, the hBMSCs were co-cultured with EC-CMVs and re-suspended in MSCM in different quantities (2.5, 5, and 10 μg). HBMSCs co-cultured with conditioned medium of hUVECs were used as a control; while untreated hBMSCs were used as a blank. The medium in all groups were exchanged every 2 to 3 days. Then the hBMSCs cultured for 7 days were subjected to alkaline phosphatase (ALP) staining. Cells were fixed with 4% (w/v) paraformaldehyde for 15 min, then washed with PBS and stained by an ALP staining kit (Beyotime).

### Alizarin red staining

To assess the mineralization, hBMSCs cultured for 14 days were stained with alizarin red S, followed by quantification of the staining intensity. Cells were fixed in 4% (w/v) paraformaldehyde and stained with 1% (w/v) AR-S (Sigma Aldrich) for 30 min. Then, the Alizarin Red-stained cells were dissolved with 10% (w/v) cetylpyridinium chloride solution at a concentration of 100 mmol/L (Sigma Aldrich) for 30 min, and the absorbance of the supernatant was then measured at a wavelength of 570 nm.

### Real-time PCR

The total RNA from cells was extracted using TRIzol reagent and the cDNA was synthesized with PrimeScript RT reagent kit (Takara Co. Japan), according to the manufacturer’s instructions. Then real-time quantitative polymerase chain (qRT-PCR) reaction was performed using SYBR Green qPCR Master Mix (Rox). Relative gene expression levels were calculated according to the cycle threshold (Ct) values relative to the endogenous housekeeping control gene (*Gapdh*) using QuantStudio Design & Analysis Desktop Software (Thermo Fisher Scientific). Glyceraldehyde-3-phosphate dehydrogenase (*Gapdh*) was used as the internal control. The primer sequences used for the experiment are listed in Table 1.

**Table 1:** Primer sequences utilized for quantitative real-time PCR analysis

Target gene	Forward sequence (5′-3′)	Reverse sequence (5′-3′)
*ColI*	AAGACGAAGACATCCCACCAATC	CAGATCACGTCATCGCACAACA
*BMP2*	TATCGCAGGCACTCAGGTCAG	GGGTTGTTTTCCCACTCGTTTC
*Runx2*	CGCCTCACAAACAACCACAG	ACTGCTTGCAGCCTTAAATGAC
*ephrinB2*	TATGCAGAACTGCGATTTCCAA	TGGGTATAGTACCAGTCCTTGTC
*Gapdh*	ACATCGCTCAGACACCATG	TGTAGTTGAGGTCAATGAAGGG

### Western blot analysis

The cells and CMVs were lysed by RIPA lysis buffer (Beyotime, China) containing a protease and phosphatase inhibitor cocktail (Thermo Fisher Scientific) on ice. After centrifugation at 12 000 *g* at 4 °C for 20 min, the total protein content was quantified by BCA protein assay kit (Beyotime, China). Then, the proteins were denatured and separated in SDS-polyacrylamide gels and were then transferred to a PVDF membrane by electrophoresis at 300 mA for 60 min. The membranes were then blocked with 5% (w/v) fat-free milk in TBST for 1 h and incubated at 4 °C overnight with the following primary antibodies: anti-Runx2 (1:1 000, Abcam), anti-BMP2 (1:1 000, Abcam), anti-VEGF (1:1 000, Cell signaling technology), anti-ephrinB2 (1:1 000, SAB), and anti-GAPDH (1:2 500, Abcam). Then, the membranes were incubated with secondary antibodies (1:5 000, Abcam) for 1 h at room temperature. The bands were then visualized by chemiluminescence using an ECL-PLUS kit (Pierce). The relative expression levels of proteins were compared through band density and the results were normalized to GAPDH.

### Immunofluorescence

HBMSCs were fixed in 4% (w/v) paraformaldehyde solution for 20 min. After being washed by PBS for 3 times, the cells were permeabilized in 0.1% (w/v) Triton X-100 for 10 min and then blocked in 5% (w/v) bovine serum albumin (BSA) for 1 h at room temperature. Subsequently, the cells were incubated overnight at 4 °C with primary antibodies anti-ColI (1:500, Abcam) and anti-Runx2 (1:200, Abcam). After washing for 3 times in PBS, the cells were incubated with secondary antibodies (1:1 000, Abcam) for 1 h in the dark at room temperature and then stained with DAPI for 15 min. Images of hBMSCs were obtained using a laser scanning confocal microscope (CLSM, Leica).

### Tube formation assays on Matrigel

The 24-well plates were coated with 250 μL Matrigel (Corning, America) per well and then placed at 37 °C for 30 min to allow Matrigel to form a gel. Then 10 × 10^4^ cells/well of hUVECs (three replicates per group) were seeded on the Matrigel (Corning, America), co-cultured with different quantities of BMSC-CMVs (2.5, 5, and 10 μg) or without BMSC-CMVs, and cultured at 37 °C in 5% CO_2_. 10 × 10^4^ hUVECs plated on the Matrigel were set as a blank control. 10 × 10^4^ hUVECs co-cultured with conditioned medium of hBMSCs were set as a control. After incubation at 37 °C for 4 and 8 h, tube structures were observed using a phase contrast microscope and then quantified with ImageJ Pro Plus 6.0 Software.

### Scratch wound assay

For the cell migration assay, hUVECs were seeded into 12-well plates and cultured to confluence. The scratch on the mono-layer was created using a P200 pipette tip, followed by washing three times with PBS to remove the detached cells. The cells were then co-cultured with BMSC-CMVs mixed in serum-free medium at different quantities. Cells cultured with serum-free medium were assigned as a blank group, while cells cultured with conditioned medium were assigned as a control. Then the cells were photographed at 6 h post-wounding and then analyzed using Image J pro plus 6.0 Software. The migration area (%) was calculated according to the formula: migration area = (M1-M0)/M0 x 100%, where M1 represents the wound area at 6 h and M0 represents the wound area at 0 h.

### Flow cytometry (FCM) analysis

HBMSCs and hUVECs were co-cultured with 5 μg of DiI-labeled EC-CMVs and 5 μg of DiO-labeled BMSC-CMVs for 12 h, respectively. Then the cells were washed three times with PBS and analyzed by flow cytometry (BD Biosciences, San Jose, CA, USA). The untreated cells were set as a negative control. The laser line wavelengths used are 488 nm for DiO and 562 nm for DiI. A minimum of 10 000 events were acquired for each sample. The evaluation of the percentages of CMVs uptake by cells was analyzed using BD FACSDiva™ software version 8.0 (*n* = 3). The collected data were further evaluated using FlowJo software (TreeStar Inc., Ashland, OR, USA).

### Preparation of CMVs with downregulated expression of ephrinB2

HBMSCs were seeded within 6-well culture dishes and transfected with the lentivirus vectors (Gnenchem, Shanghai, China) for knockdown of *EFNB2* at a multiplicity of infection (MOI) of 50. The sequence of the shRNA was as follows: 5′-CCGGCGACAACAAGTCCCTTTGTAACTCGAGTTACAAAGGGACTTGTTGTCGTTTTTG-3′. Then, the transfected cells were expanded for the preparation of BMSC-CMVs using the previous method. The shRNA transfection efficiency was determined by Real-time PCR and western blot analysis.

### Animal surgery and treatment

Nine 4 weeks old male BALB/c nude mice were purchased from the Beijing Vital River Laboratory Animal Technology Co., Ltd. (Beijing, China) and housed in a pathogen-free facility. Nude mice were injected subcutaneously with 5 × 10^6^ hUVECs mixed with BMSC-CMVs or BMSC-CMVs (*EFNB2*-shRNA) produced by 5 × 10^6^ hBMSCs and hBMSCs (*EFNB2*-shRNA) mixed in Matrigel, respectively. Mice injected with 5 × 10^6^ hUVECs were assigned as the blank control. Each group included 6 mice: (i) hUVECs; (ii) hUVECs + BMSC-CMVs in; (iii) hUVECs + BMSC-CMVs (*EFNB2*-shRNA).

Twenty-four 6 to 8 weeks old male SD rats were purchased from the Beijing Vital River Laboratory Animal Technology Co., Ltd. (Beijing, China) and maintained in a pathogen-free facility. After being anesthetized by intraperitoneal administration of 100 mg/kg pentobarbital sodium (Sigma-Aldrich), a full thickness bone defect of 5 mm in diameter was prepared in each rat cranium. The rats were randomly allocated into 4 groups: (i) Matrigel; (ii) BMSC-CMVs produced by 5 × 10^6^ hBMSCs mixed in Matrigel; (iii) EC-CMVs produced by 5 × 10^6^ hUVECs mixed in Matrigel; (iv) BMSC-CMVs + BMSC-CMVs produced by 5 × 10^6^ hBMSCs and 5 × 10^6^ hUVECs respectively mixed in Matrigel. After 4 and 8 weeks, the skull samples were collected and fixed with 4% (w/v) paraformaldehyde for subsequent micro-CT scanning and histological analysis.

### Micro-computed tomography (CT)

Micro-CT was performed to analyze new bone formation within the defects. The harvested calvaria samples were collected and examined using micro-CT as previously described.^37^ The samples were scanned using a micro-computed tomographic system (GANTRY- STD CT 3121; Siemens, Knoxville, TN). After three-dimensional (3D) visualization, bone volume analyses of the samples were carried out on the region of interest (ROI) using a microtomographic analysis software (Tomo NT; Skyscan, Belgium).

### Hematoxylin and eosin (H&E) and Masson’s trichrome staining

After scanning by micro-CT, the samples were collected for histological analysis. The samples were fixed in 4% (w/v) paraformaldehyde solution, decalcified with 5% (w/v) EDTA and embedded in paraffin. Then the embedded samples were cut into 4-μm-thick sections (Leica Instruments GmbH, Hubloch, Germany) and sections from the defect region were stained with hematoxylin and eosin (H&E) according to the manufacturer’s protocol. Then histological sections of the tissues were processed with Masson’s trichrome staining to evaluate the degree of collagen maturity within the defect sites. The stained sections were imaged under an optical microscope (Olympus, Tokyo, Japan).

### Immunohistochemistry

The paraffin-embedded samples were cut into 5 μm thick sections for immunohistochemistry analysis. The sections were incubated with antibodies against CD31 (Abcam) overnight at 4 °C, followed by incubation with secondary antibody (Servicebio). Immunoreactivity for CD31 was assessed by evaluating the positive area percentage values with Image J 2.0.0 software.

### Statistical analysis

All experiments were performed with at least 3 replicates per group and all data were presented as mean ± SD. Comparisons between groups were evaluated by one-way ANOVA. Statistical analysis was conducted using PRISM software, version 7 and the threshold of statistical significance was set at *P* < 0.05.

### Supplementary information

Cell membrane vesicles derived from hBMSCs and hUVECs enhance bone regeneration

Data set 1

## Data Availability

All relevant data supporting the key findings of this study are available within the article. Raw sequencing data are available from the corresponding author on reasonable request.

## References

[1] Schott NG, Friend NE, Stegemann JP (2021). Coupling osteogenesis and vasculogenesis in engineered orthopedic tissues. Tissue Eng. B Rev..

[2] Eshkar-Oren I (2009). The forming limb skeleton serves as a signaling center for limb vasculature patterning via regulation of Vegf. Development.

[3] Maes C (2010). Osteoblast precursors, but not mature osteoblasts, move into developing and fractured bones along with invading blood vessels. Dev. Cell.

[4] Bolander J (2017). Healing of a large long-bone defect through serum-free in vitro priming of human periosteum-derived cells. Stem Cell Rep..

[5] Queiroz A (2021). Therapeutic potential of periodontal ligament stem cells. World J. Stem Cells.

[6] Bayat H (2019). Osteogenic differentiation of follicular stem cells on nano-Saghez scaffold containing BMP2. J. Orthop. Surg. Res..

[7] Einhorn TA, Gerstenfeld LC (2015). Fracture healing: mechanisms and interventions. Nat. Rev. Rheumatol..

[8] Bruder SP (1998). Bone regeneration by implantation of purified, culture-expanded human mesenchymal stem cells. J. Orthop. Res..

[9] Liu Y (2012). Vasculogenic and osteogenesis-enhancing potential of human umbilical cord blood endothelial colony-forming cells. Stem Cells.

[10] Fiedler J, Brill C, Blum WF, Brenner RE (2006). IGF-I and IGF-II stimulate directed cell migration of bone-marrow-derived human mesenchymal progenitor cells. Biochem. Biophys. Res. Commun..

[11] Liu Y, Chan JK, Teoh SH (2015). Review of vascularised bone tissue-engineering strategies with a focus on co-culture systems. J. Tissue Eng. Regen. Med..

[12] Lee HY, Hong IS (2017). Double-edged sword of mesenchymal stem cells: cancer-promoting versus therapeutic potential. Cancer Sci..

[13] Herberts CA, Kwa MS, Hermsen HP (2011). Risk factors in the development of stem cell therapy. J. Transl. Med..

[14] Kolios G, Moodley Y (2013). Introduction to stem cells and regenerative medicine. Respiration.

[15] Terrovitis JV, Smith RR, Marbán E (2010). Assessment and optimization of cell engraftment after transplantation into the heart. Circ. Res..

[16] Phinney DG, Pittenger MF (2017). Concise review: MSC-derived exosomes for cell-free therapy. Stem Cells.

[17] Pick H (2005). Investigating cellular signaling reactions in single attoliter vesicles. J. Am. Chem. Soc..

[18] Wang D (2021). Cell membrane vesicles with enriched CXCR4 display enhances their targeted delivery as drug carriers to inflammatory sites. Adv. Sci..

[19] Gomzikova MO (2020). Immunosuppressive properties of cytochalasin B-induced membrane vesicles of mesenchymal stem cells: comparing with extracellular vesicles derived from mesenchymal stem cells. Sci. Rep-Uk.

[20] Peng LH (2015). Cell membrane capsules for encapsulation of chemotherapeutic and cancer cell targeting in vivo. Acs Appl. Mater. Inter..

[21] Schoenenberger CA, Bischler N, Fahrenkrog B, Aebi U (2002). Actin’s propensity for dynamic filament patterning. FEBS Lett..

[22] Gilazieva Z (2022). Comparative analysis of natural and cytochalasin B-induced membrane vesicles from tumor cells and mesenchymal stem cells. Curr. Issues Mol. Biol..

[23] Chulpanova DS (2023). Cytochalasin B-induced membrane vesicles from TRAIL-overexpressing mesenchymal stem cells induce extrinsic pathway of apoptosis in breast cancer mouse model. Curr. Issues Mol. Biol..

[24] Cao C (2018). Bidirectional juxtacrine ephrinB2/Ephs signaling promotes angiogenesis of ECs and maintains self-renewal of MSCs. Biomaterials.

[25] Salomon C (2013). Exosomal signaling during hypoxia mediates microvascular endothelial cell migration and vasculogenesis. PLoS One.

[26] Bian S (2014). Extracellular vesicles derived from human bone marrow mesenchymal stem cells promote angiogenesis in a rat myocardial infarction model. J. Mol. Med..

[27] Zhang L (2020). Exosomes from bone marrow mesenchymal stem cells enhance fracture healing through the promotion of osteogenesis and angiogenesis in a rat model of nonunion. Stem Cell Res. Ther..

[28] Peng LH (2015). Cell membrane capsules for encapsulation of chemotherapeutic and cancer cell targeting in vivo. ACS Appl. Mater. Interfaces.

[29] Mao Z (2011). Cells as factories for humanized encapsulation. Nano Lett..

[30] Gomzikova M, Kletukhina S, Kurbangaleeva S, Rizvanov A (2018). Evaluation of cytochalasin B-induced membrane vesicles fusion specificity with target cells. BioMed Res. Int..

[31] Rüster B (2006). Mesenchymal stem cells display coordinated rolling and adhesion behavior on endothelial cells. Blood.

[32] Ramasamy SK, Kusumbe AP, Wang L, Adams RH (2014). Endothelial Notch activity promotes angiogenesis and osteogenesis in bone. Nature.

[33] Diomede F (2020). Functional relationship between osteogenesis and angiogenesis in tissue regeneration. Int. J. Mol. Sci..

[34] Gomzikova MO (2020). Immunosuppressive properties of cytochalasin B-induced membrane vesicles of mesenchymal stem cells: comparing with extracellular vesicles derived from mesenchymal stem cells. Sci. Rep..

[35] Martens S, McMahon HT (2008). Mechanisms of membrane fusion: disparate players and common principles. Nat. Rev. Mol. Cell Biol..

[36] Zhidkova OV, Andreeva ER, Buravkova LB (2018). Endothelial cells modulate differentiation potential and mobility of mesenchymal stromal cells. Bull. Exp. Biol. Med..

[37] Zhang X (2016). Nanocomposite membranes enhance bone regeneration through restoring physiological electric microenvironment. ACS Nano.

