# Supplementary material for: Cell membrane vesicles derived from hBMSCs and hUVECs enhance bone regeneration
Source: Bone Res. 2024 Apr 9;12:23. doi: 10.1038/s41413-024-00325-9 (PMC11003965; doi:10.1038/s41413-024-00325-9)
Supplement: Supplementary file 1 — Cell membrane vesicles derived from hBMSCs and hUVECs enhance bone regeneration [file 41413_2024_325_MOESM1_ESM.docx]

Supporting Information

**Cell membrane vesicles derived from hBMSCs and hUVECs enhance bone regeneration**

Dandan Wang, Yaru Guo, Boon Chin Heng, Xuehui Zhang, Yan Wei, Ying He, Mingming Xu, Bin Xia & Xuliang Deng

**Table of contents**

**Supplementary tables**

Supplementary table 1：Gene names of EC-CMVs that relate to osteogenesis.

Supplementary table 2：Gene names of BMSC-CMVs that relate to angiogenesis.

**Supplementary Figures:**

Supplementary Figure 1: CD90 immunocytochemical staining images of human umbilical vein endothelial cells.

Supplementary Figure 2: CD31 immunocytochemical staining images of bone marrow mesenchymal stem cells.

Supplementary Figure 3: Changes in the fibrous cytoskeletal framework of hBMSCs (a) and hUVECs (b) after treatment with CB for 30 minutes. The formation of spherical cell capsules was observed around the cells.

Supplementary Figure 4: Expression of Runx2 and BMP2 at the protein level was determined by western blot analysis (***P*＜0.01, n=3).

Supplementary Figure 5: Expression of BMP2 and VEGF at the protein level was determined by western blot analysis (***P*＜0.01, n=3).

Supplementary Figure 6: Expression of VEGF at the protein level was determined by western blot analysis (***P*＜0.01, n=3).

Supplementary Figure 7: Expression of ephrinB2 at the protein level was determined by western blot analysis (****P*＜0.001, n=3).

Supplementary Figure 8: Realtime-PCR and western blot analysis showed that ephrinB2 gene expression levels and protein expression levels of transfected cells were downregulated after lentivirus transfection (****P*＜0.001, n=3)

Supplementary Figure 9: Representative analysis of tube formation at 4 and 8 h after hUVECs were co-cultured with BMSC-CMVs and BMSC-CMVs (*EFNB2*-shRNA).

Supplementary Figure 10: BMSC-CMVs (*EFNB2*-shRNA) fluorescence emission (green) in hUVECs were lower, as compared to BMSC-CMVs fluorescence emission in Fig. 5e.

Supplementary Figure 11: Representative images of Matrigel plugs with hUVECs, hUVECs+BMSC-CMVs and hUVECs+BMSC-CMVs (*EFNB2*-shRNA) at 4 days after subcutaneous injection in nude mice.

Supplementary Figure 12: [KEGG (Kyoto Encyclopedia of Genes and Genomes](https://www.genome.jp/kegg/)) analysis of BMSC-CMVs.

Supplementary Figure 13: [KEGG (Kyoto Encyclopedia of Genes and Genomes](https://www.genome.jp/kegg/)) analysis of EC-CMVs.

Supplementary Figure 14: G.O. (Gene Ontology) analysis with FunRich software of identified differentially expressed proteins of BMSC-CMVs: Biological Process, Cellular component, and Molecular Function.

Supplementary Figure 15: G.O. (Gene Ontology) analysis with FunRich software of identified differentially expressed proteins of EC-CMVs: Biological Process, Cellular component and Molecular Function.

Supplementary Figure 16: CD31 and OCN area% of immunohistochemical staining images in Figure 6c, d were quantified using Image J software.

**Table 1.** Gene names of EC-CMVs that relate to osteogenesis.

***
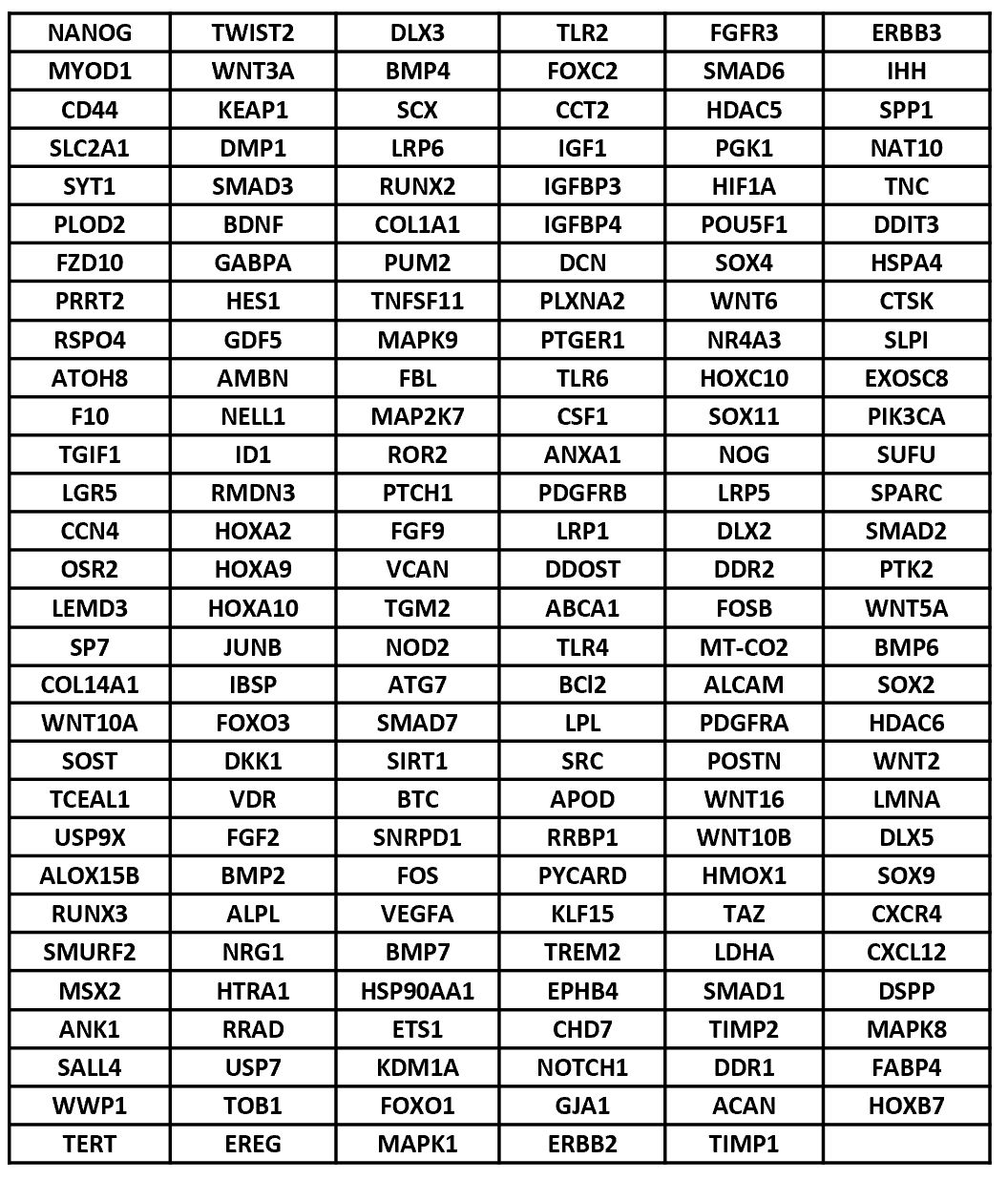
***

**Table 2.** Gene names of BMSC-CMVs that relate to angiogenesis.

**
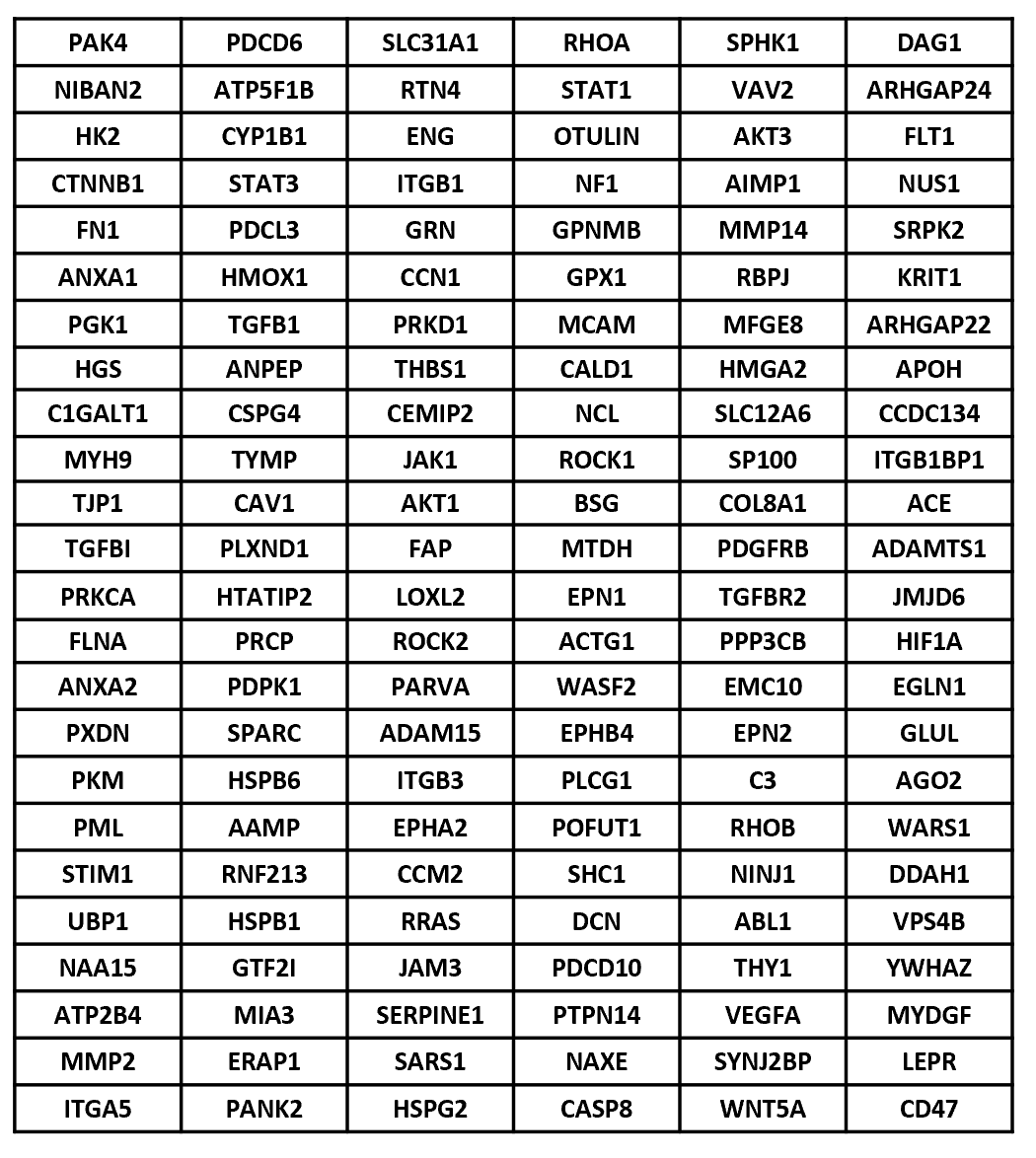
**


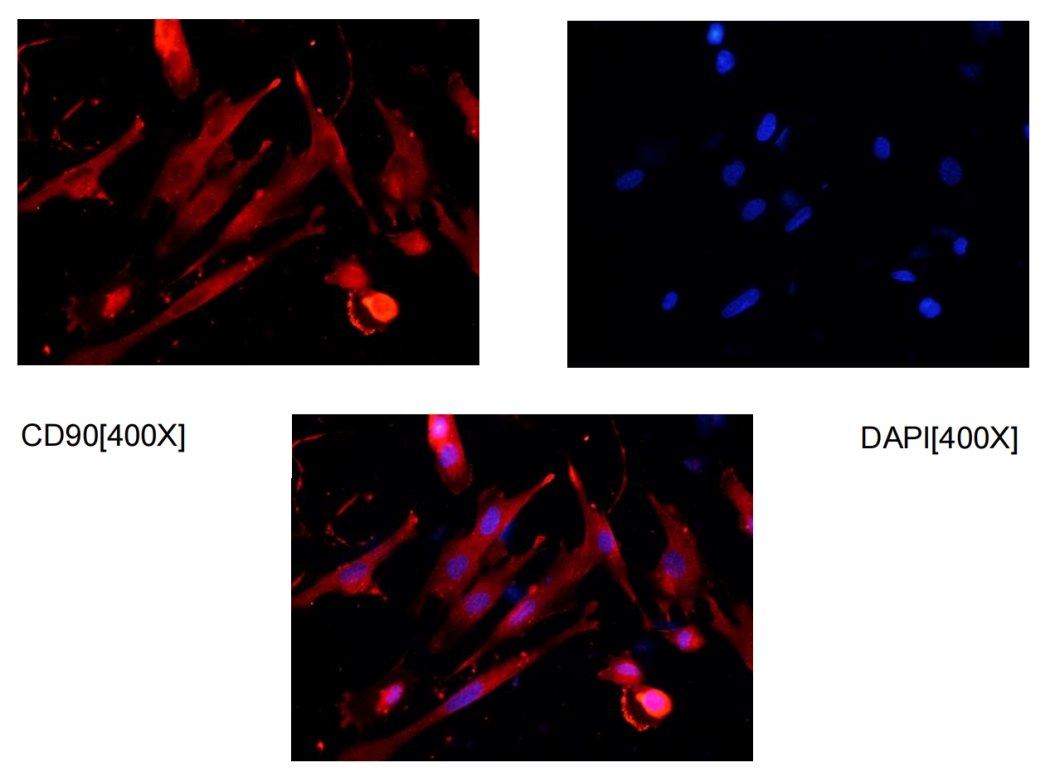


**Supplementary Figure 1:** CD90 immunocytochemical staining images of human umbilical vein endothelial cells.


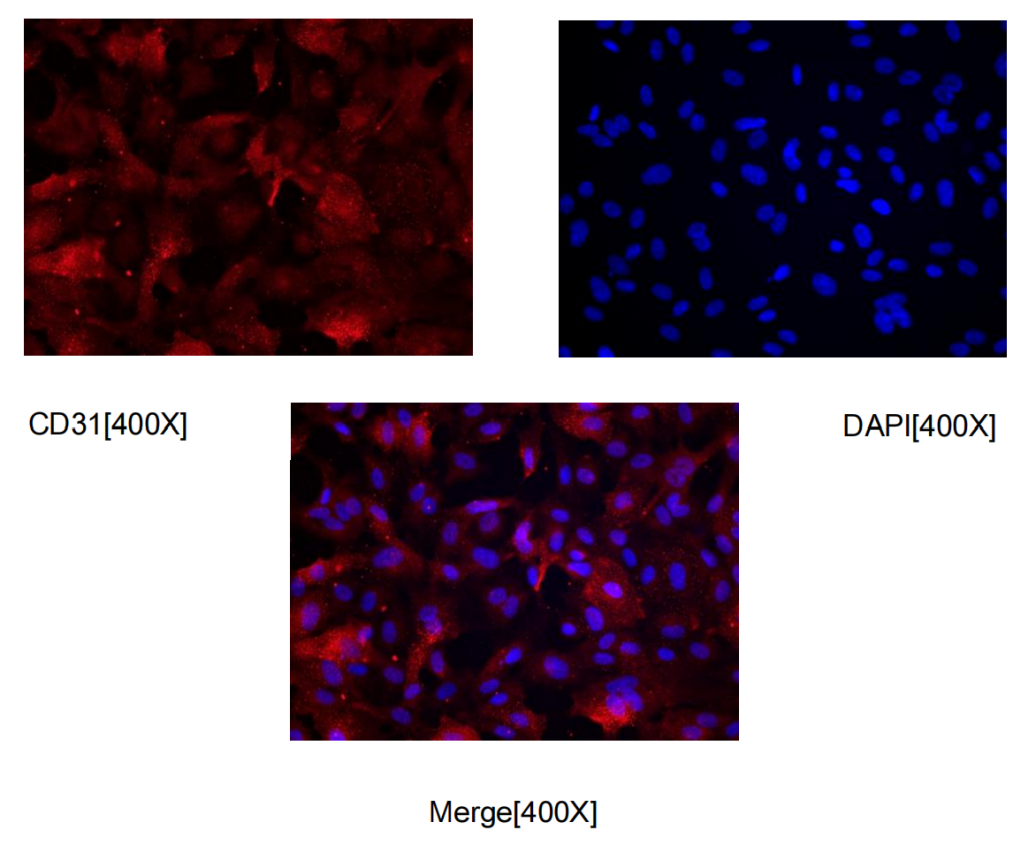


**Supplementary Figure 2:** CD31 immunocytochemical staining images of bone marrow mesenchymal stem cells.

**
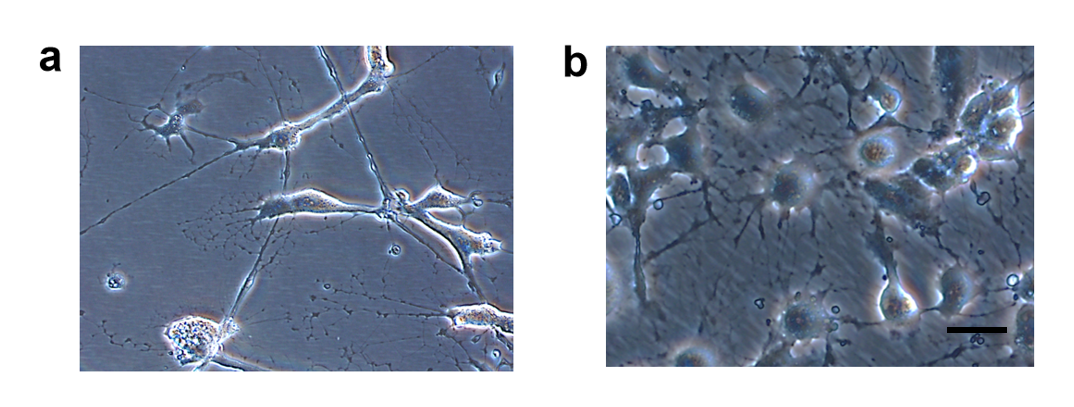
**

**Supplementary Figure 3:** Changes in the fibrous framework of hBMSCs (a) and hUVECs (b) after treatment with CB for 30 minutes. The formation of spherical cell capsules was observed around the cells. The scale bar = 50 μm.

**
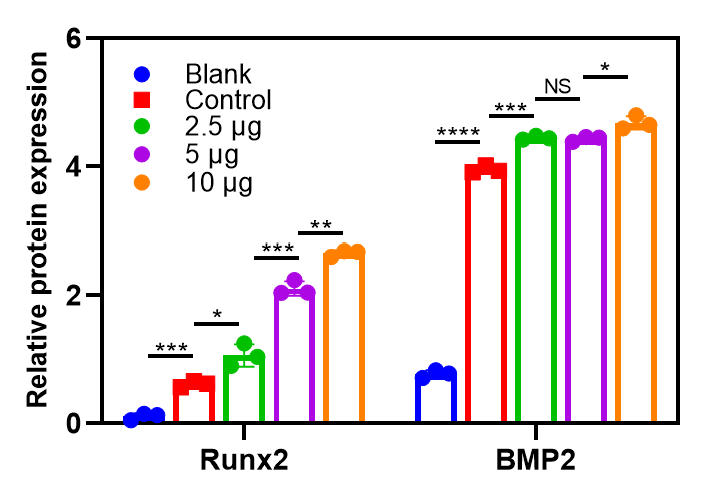
**

**Supplementary Figure 4:** Expression of Runx2 and BMP2 at the protein level was determined by western blot analysis (**P*＜0.05, ***P*＜0.01, ****P*＜0.001, *****P*＜0.0001, n=3).


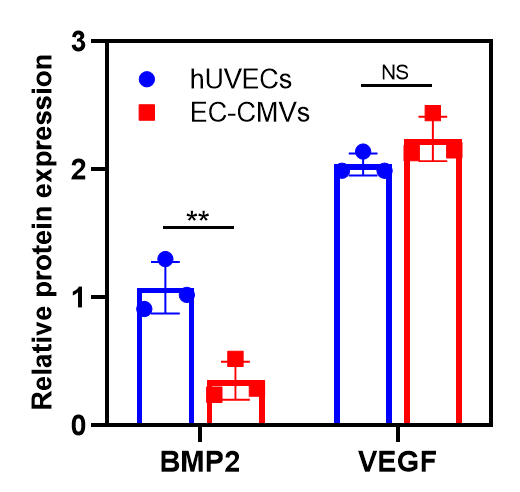
**Supplementary Figure 5:** Expression of BMP2 and VEGF at the protein level was determined by western blot analysis (***P*＜0.01, n=3).


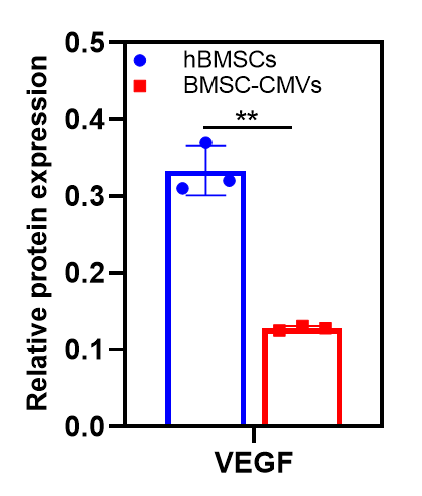
**Supplementary Figure 6:** Expression of VEGF at the protein level was determined by western blot analysis (***P*＜0.01, n=3).


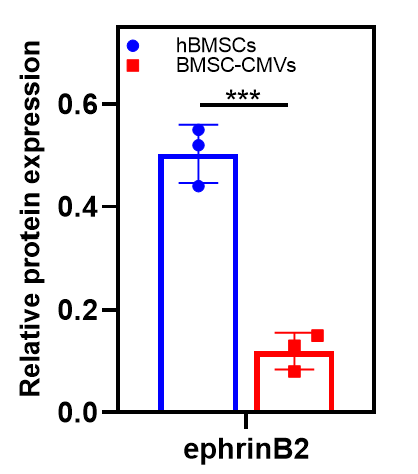


**Supplementary Figure 7:** Expression of ephrinB2 at the protein level was determined by western blot analysis (****P*＜0.001, n=3).


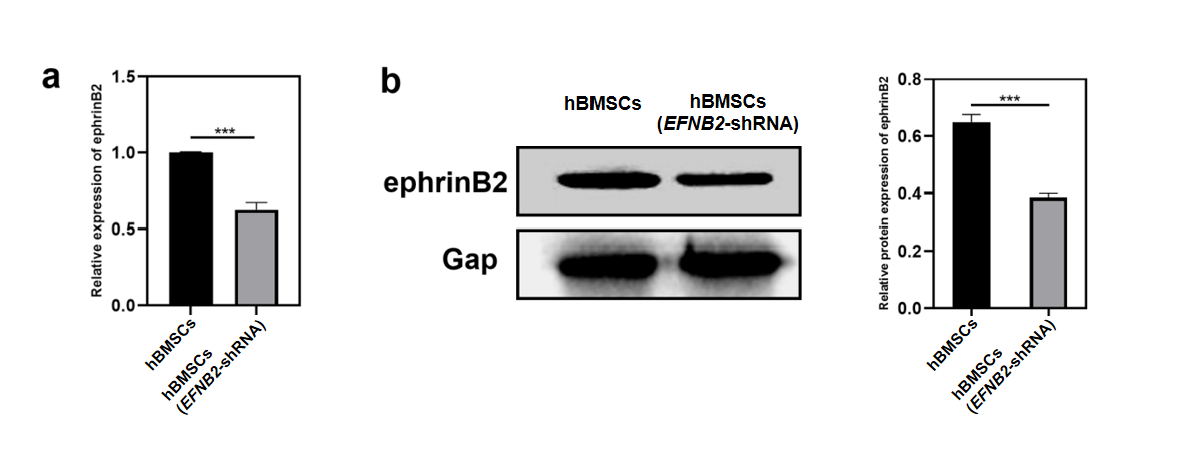


**Supplementary Figure 8:** Realtime-PCR and western blot analysis showed that ephrinB2 gene expression levels and protein expression levels of transfected cells were downregulated after lentivirus transfection (****P*＜0.001, n=3)


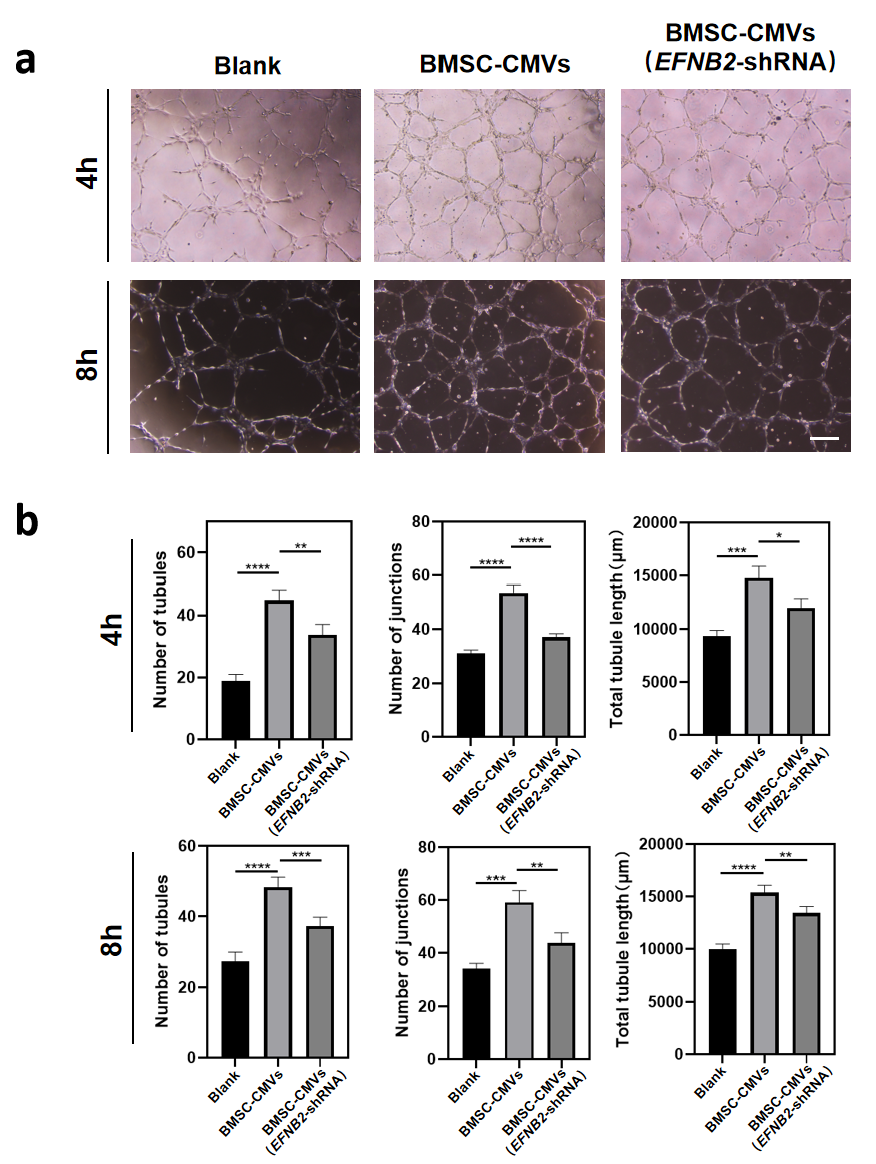
**Supplementary Figure 9**: Representative analysis of tube formation at 4 and 8 h after hUVECs were co-cultured with BMSC-CMVs and BMSC-CMVs (*EFNB2*-shRNA). Number of tubules, number of junctions and total tubule lengths in each group at 4 and 8 h in Fig. 5h were quantified using Image Pro Plus 6.0 software (**P*＜0.05, ***P*＜0.01, ****P*＜0.001, *****P*＜0.0001, n=3).


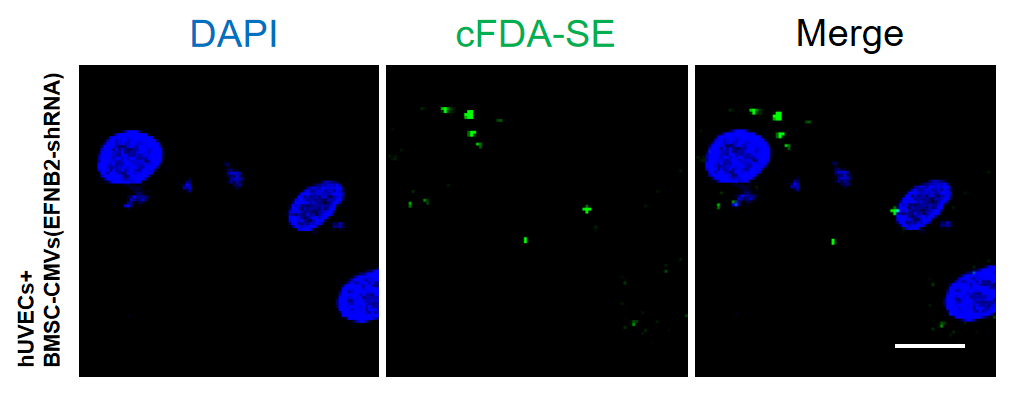
**Supplementary Figure 10**: BMSC-CMVs (*EFNB2*-shRNA) fluorescence emission (green) in hUVECs were lower, as compared to BMSC-CMVs fluorescence emission in Fig. 5f. The scale bar = 20 μm.


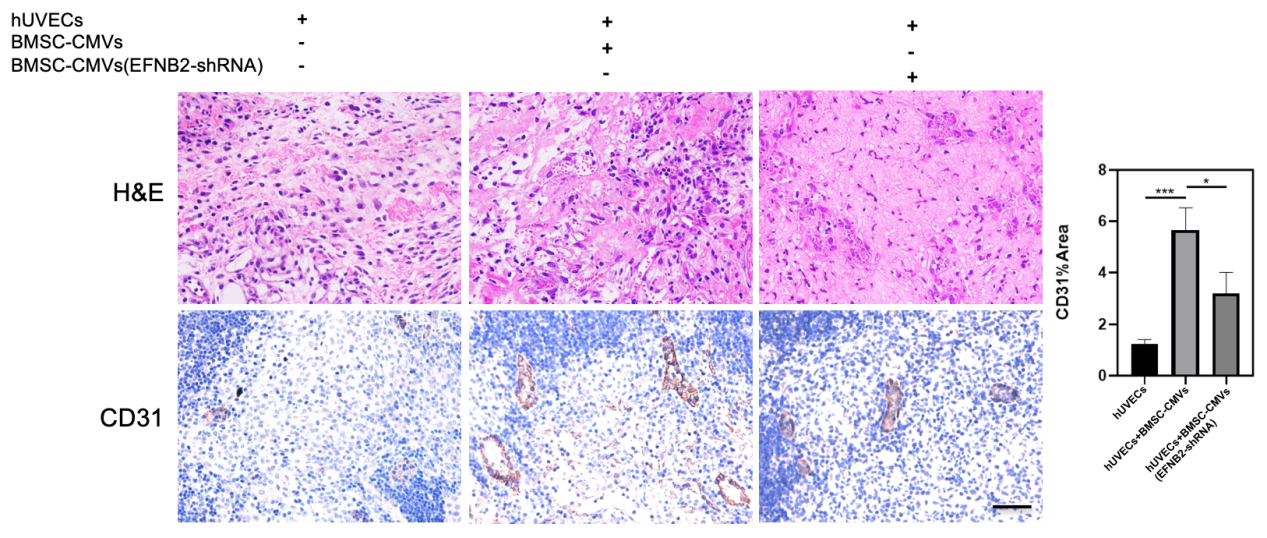
**Supplementary Figure 11**: Representative images of Matrigel plugs with hUVECs, hUVECs+BMSC-CMVs and hUVECs+BMSC-CMVs (*EFNB2*-shRNA) at 4 days after subcutaneous injection in nude mice. Hematoxylin/eosin staining and immunohistochemistry staining images for detection of CD31 expression within the three groups. The scale bar = 100 µm. CD31 area% were quantified using Image J software (**P*＜0.05, ****P*＜0.001, n=6).

## **Supplementary Figure 1**
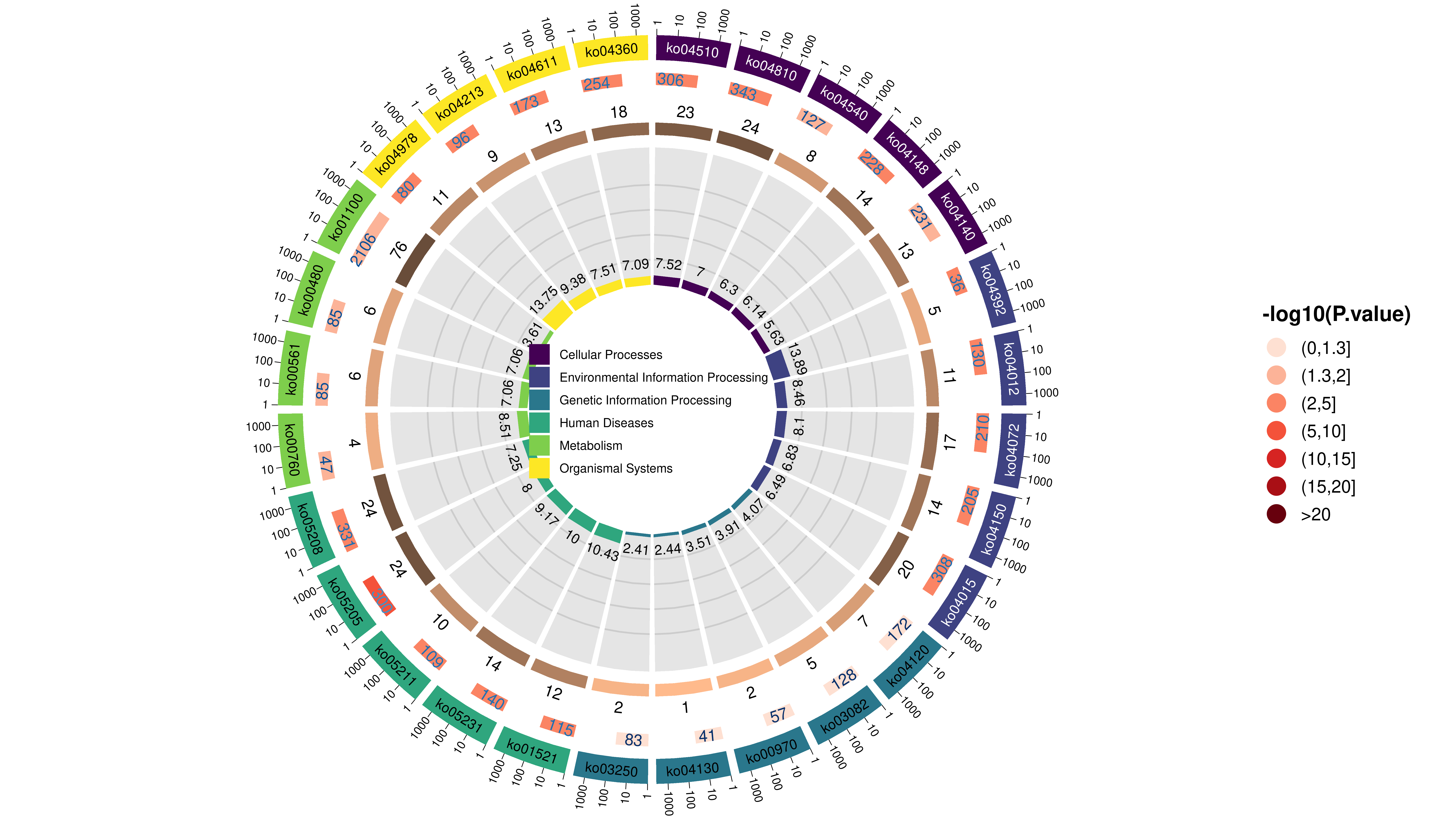
**2**: [KEGG (Kyoto Encyclopedia of Genes and Genomes](https://www.genome.jp/kegg/)) analysis of BMSC-CMVs.


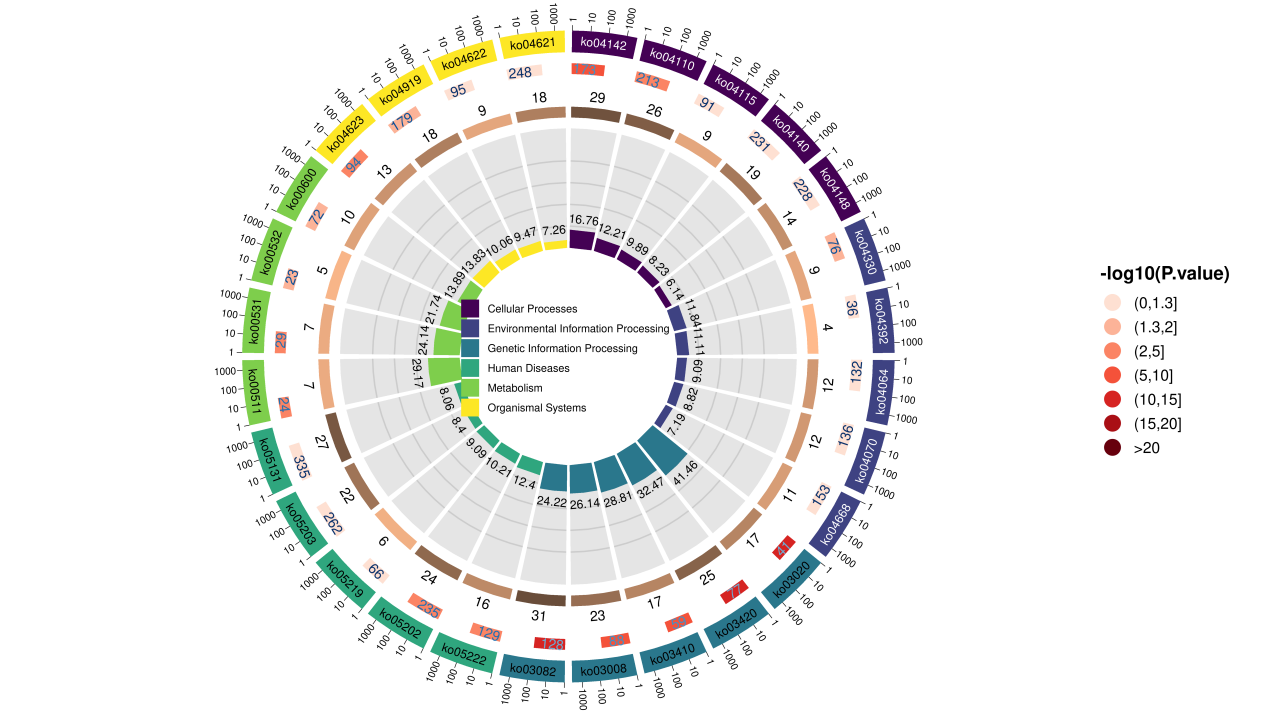


## **Supplementary Figure 1
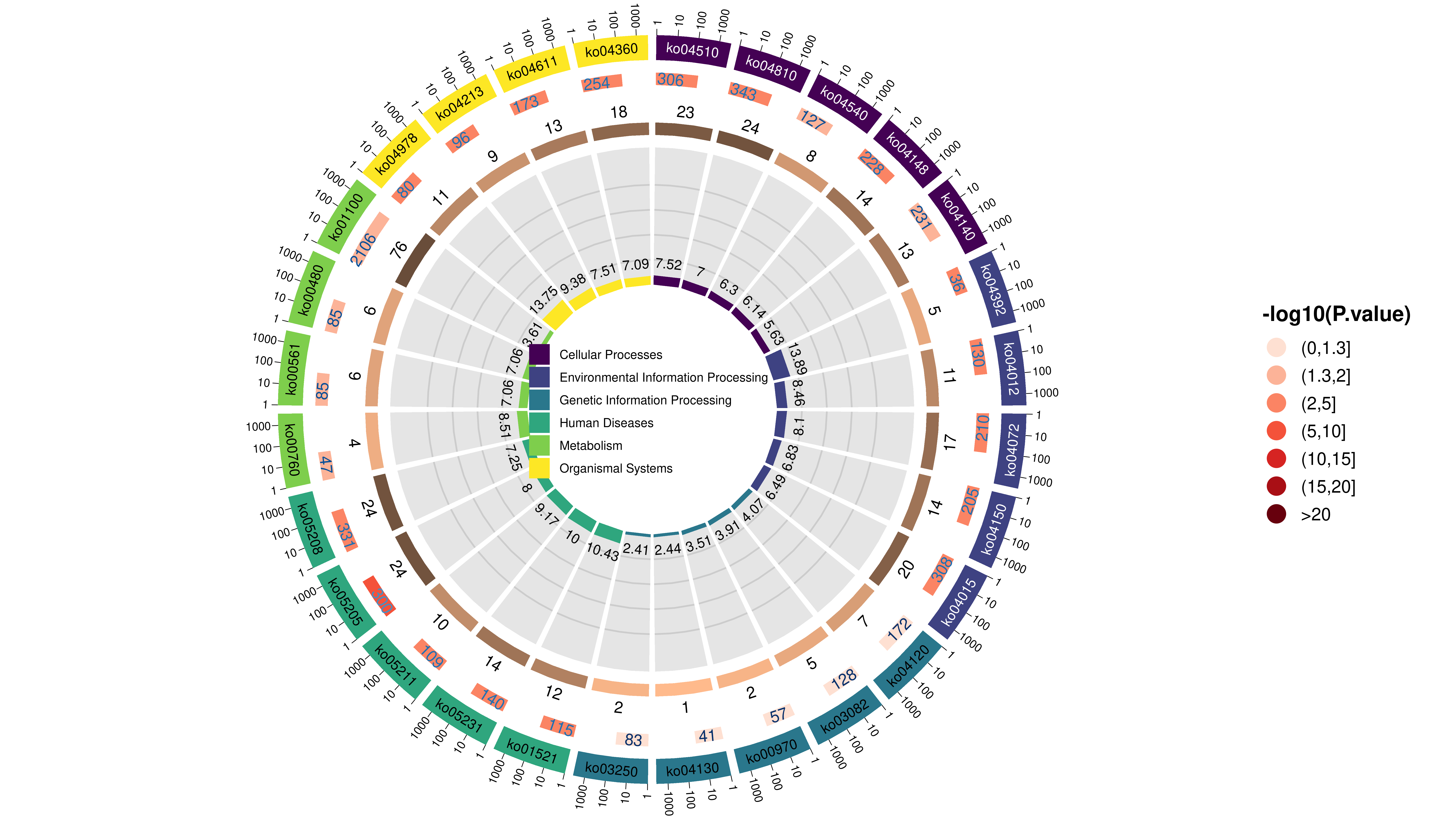
3**: [KEGG (Kyoto Encyclopedia of Genes and Genomes](https://www.genome.jp/kegg/)) analysis of EC-CMVs.


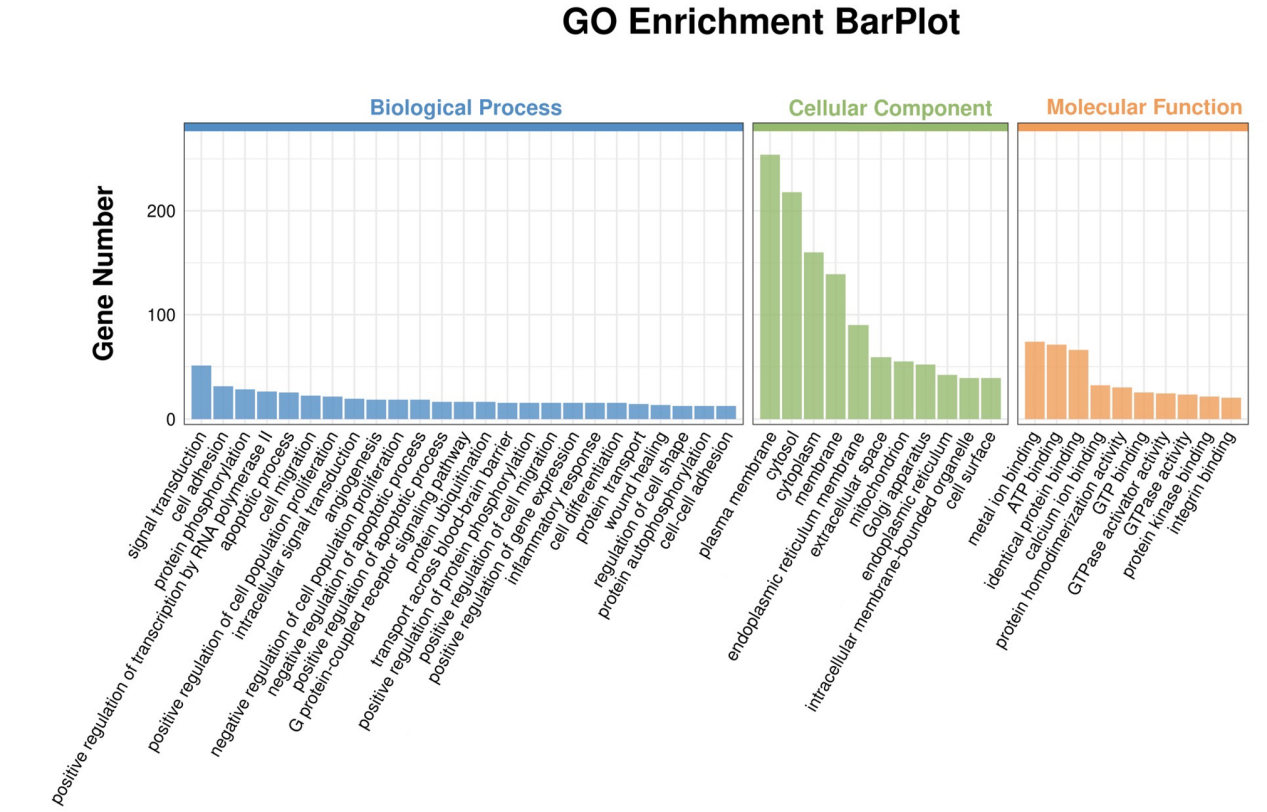
**Supplementary Figure 14**: G.O. (Gene Ontology) analysis with FunRich software of identified differentially expressed proteins of BMSC-CMVs: Biological Process, Cellular component, and Molecular Function.

**
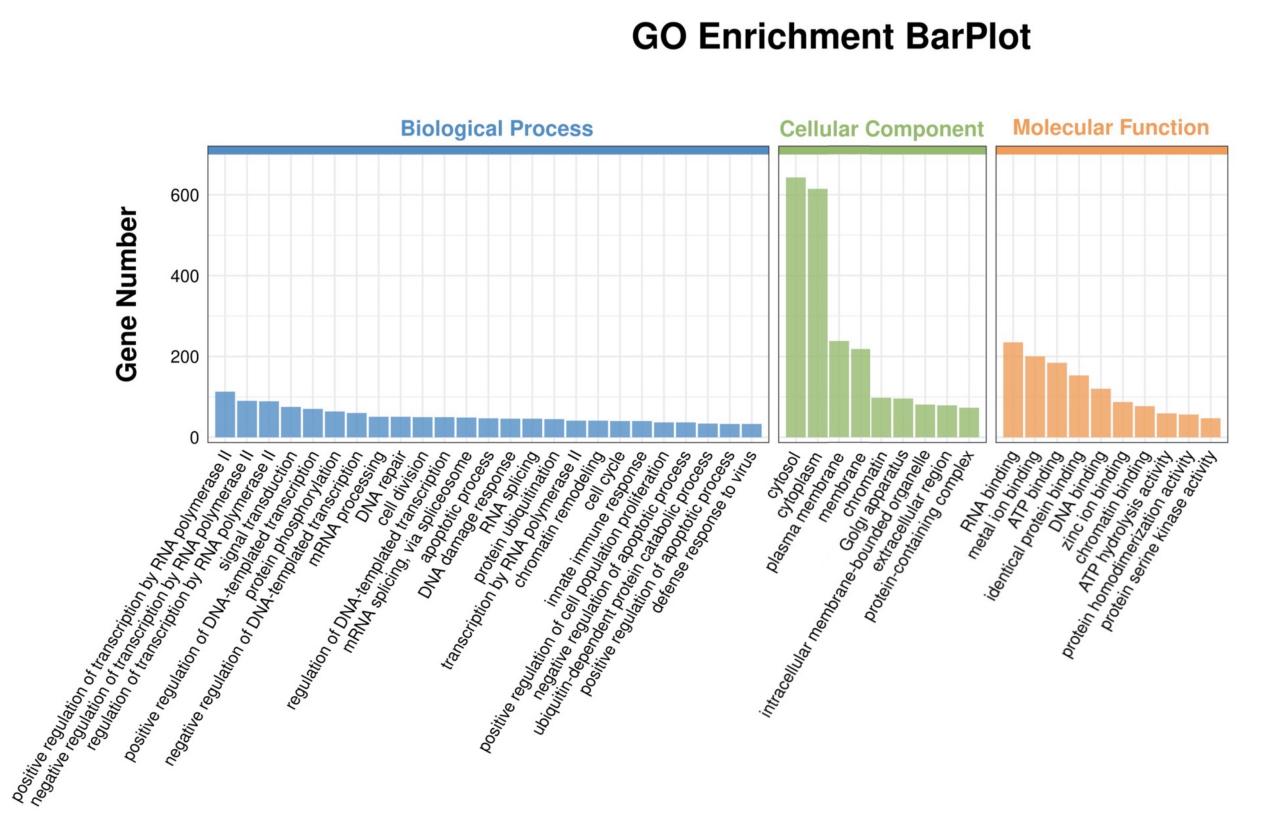
Supplementary Figure 15**: G.O. (Gene Ontology) analysis with FunRich software of identified differentially expressed proteins of EC-CMVs: Biological Process, Cellular component and Molecular Function.


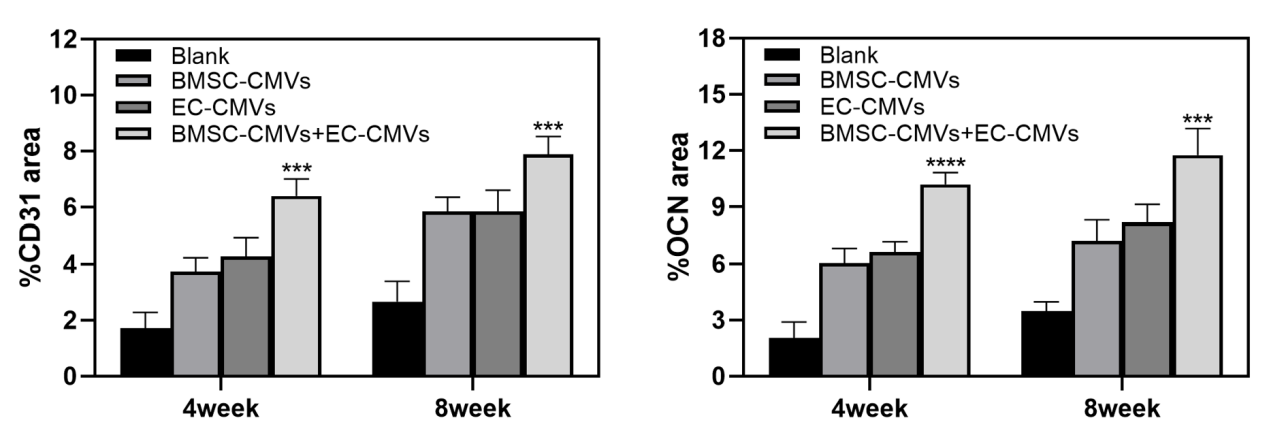
**Supplementary Figure 16**: CD31 and OCN area% of immunohistochemical staining images in Figure 6d were quantified using Image J software (****P*＜0.001, *****P*＜0.0001 compared with the blank group, n=6).
